# Bacterial Toxin‐Triggered Release of Antibiotics from Capsosomes Protects a Fly Model from Lethal Methicillin‐Resistant *Staphylococcus aureus* (MRSA) Infection

**DOI:** 10.1002/adhm.202200036

**Published:** 2022-05-11

**Authors:** Renée L. Tonkin, Anna Klöckner, Adrian Najer, Carolina J. Simoes da Silva, Cécile Echalier, Marc S. Dionne, Andrew M. Edwards, Molly M. Stevens

**Affiliations:** ^1^ Department of Materials Department of Bioengineering and Institute of Biomedical Engineering Imperial College London London SW7 2AZ UK; ^2^ MRC Centre for Molecular Bacteriology and Infection Imperial College London London SW7 2AZ UK; ^3^ Department of Life Sciences Imperial College London London SW7 2AZ UK; ^4^ Hybrid Technology Hub‐Centre of Excellence Institute of Basic Medical Sciences University of Oslo Oslo 0315 Norway

**Keywords:** antibiotic delivery, capsosomes, microparticles, MRSA, vancomycin

## Abstract

Antibiotic resistance is a severe global health threat and hence demands rapid action to develop novel therapies, including microscale drug delivery systems. Herein, a hierarchical microparticle system is developed to achieve bacteria‐activated single‐ and dual‐antibiotic drug delivery for preventing methicillin‐resistant *Staphylococcus aureus* (MRSA) bacterial infections. The designed system is based on a capsosome structure, which consists of a mesoporous silica microparticle coated in alternating layers of oppositely charged polymers and antibiotic‐loaded liposomes. The capsosomes are engineered and shown to release their drug payloads in the presence of MRSA toxins controlled by the Agr quorum sensing system. MRSA‐activated single drug delivery of vancomycin and synergistic dual delivery of vancomycin together with an antibacterial peptide successfully kills MRSA in vitro. The capability of capsosomes to selectively deliver their cargo in the presence of bacteria, producing a bactericidal effect to protect the host organism, is confirmed in vivo using a *Drosophila melanogaster* MRSA infection model. Thus, the capsosomes serve as a versatile multidrug, subcompartmentalized microparticle system for preventing antibiotic‐resistant bacterial infections, with potential applications to protect wounds or medical device implants from infections.

## Graphical abstract

Bacterial toxin‐activated capsosomes are employed for single and synergistic dual delivery of antimicrobial drugs for the prevention of methicillin‐resistant *Staphylococcus aureus* (*S. aureus*) (MRSA) infections. The toxin‐triggered release mechanism is characterized and the bactericidal efficacy of the antibiotic‐loaded capsosomes is demonstrated in vitro and in an in vivo fly infection model of lethal MRSA infection.

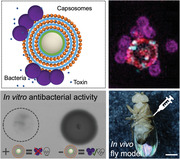

## 1 Introduction

The worldwide transmission of infectious diseases can have devastating health, social, and economic effects, as evidenced by the COVID‐19 pandemic. Antibiotic resistance represents a similar, although more gradually developing, global health threat and hence demands rapid action to develop novel and innovative therapies to tackle bacterial infections. Methicillin‐resistant *Staphylococcus aureus* (MRSA) strains are of particular concern since they are already resistant to several widely used antibiotics and can cause a variety of diseases including healthcare associated infections.^[^ ^1^, ^2^, ^3^ ^]^ Current treatment regimens include drugs such as vancomycin which is associated with toxicity and efficacy issues.^[^ ^4^ ^]^ Therefore, novel antibiotic therapies are needed to deliver antibiotics locally in the early stages of an infection in a controlled way to increase efficacy and lower toxicity.

Micron‐scale drug delivery systems are attractive approaches for localized antibiotic delivery in the treatment of *S. aureus* infections^[^ ^5^, ^6^ ^]^ and for improving multidrug therapies by modulating the release kinetics of the different compounds.^[^ ^7^ ^]^ Controlled and local release of antibiotics is considered an option to reduce systemic toxicity, such as nephrotoxicity and ototoxicity.^[^ ^6^ ^]^ Furthermore, both single‐ and multidrug delivery systems can be designed to trigger local antibiotic release in response to stimuli associated with infection conditions, including lowered pH, membrane‐damaging toxins, and upregulation of host enzyme expression.^[^ ^8^, ^9^, ^10^, ^11^, ^12^, ^13^, ^14^, ^15^, ^16^ ^]^ *S. aureus* produces large quantities of toxins which damage eukaryotic cell membranes via pore‐formation (e.g., *α*‐toxin), lipase activity (e.g., *β*‐toxin), or surfactant‐like mechanisms (e.g., phenol soluble modulins (PSMs)).^[^ ^17^ ^]^ Therefore, bacteria‐activated release of antibiotics has been achieved by including a gating lipid component in the design of the drug delivery systems.^[^ ^10^, ^11^, ^12^, ^13^, ^14^ ^]^ Unlike systemic antibiotic therapy, these toxin triggerable drug delivery systems could help to protect a patient's microbiota by restricting antibiotic release to the time and site of an infection.

**Figure 1:**
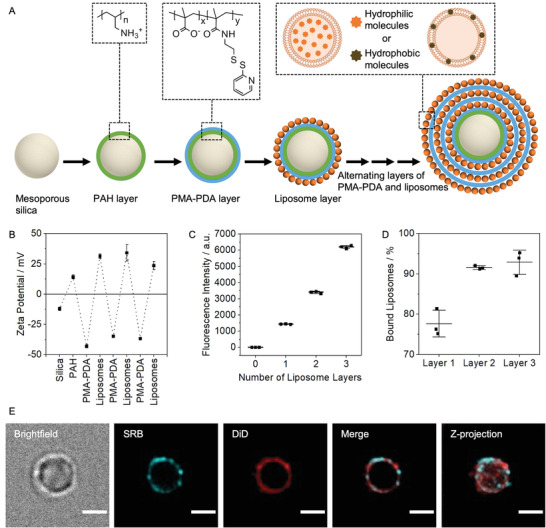
Multilayer assembly of capsosomes. A) Schematic describing layer‐by‐layer (LbL) capsosome assembly process. B) Alternating zeta potential of capsosomes after adsorption of each layer of polymer or liposomes (*N* = 3 independent samples, *n* = 3 technical repeats). C) Fluorescence intensity after adsorption of one, two, or three layers of DiD‐loaded liposomes (*N* = 3 independent samples, *n* = 1 technical repeats). D) Percentage of liposomes bound to capsosomes after adsorption of the first, second, and third layers, calculated by measuring the concentration of liposomes remaining in the supernatant after adsorption and subtracting that from the initial liposome concentration (*N* = 3 independent samples, *n* = 3 technical repeats). E) Deconvolved widefield fluorescence micrographs showing a capsosome containing SRB‐loaded liposomes in layers one and two, representing a hydrophilic drug loading, and DiD‐loaded liposomes in layer three, representing a hydrophobic drug loading. Scale bars: 2 µm. More images shown in Figure S5, Supporting Information. PAH: poly(allylamine hydrochloride), PMA‐PDA: poly(methacrylic acid) functionalized with pyridine dithioethylamine, SRB: sulforhodamine B, DiD: 1,1‐dioctadecyl‐3,3,3,3‐tetramethylindodicarbocyanine. Data shown as mean ± s.d.

Advanced forms of hierarchical microassemblies are being employed to improve multidrug delivery in a range of diseases.^[^ ^18^ ^]^ One example of these are capsosomes, which have previously been applied for the delivery of anticancer drugs.^[^ ^19^, ^20^ ^]^ Capsosomes comprise a multilayer assembly of alternating layers of oppositely charged polymers and liposomes formed on a solid, mesoporous, or soft material core.^[^ ^21^, ^22^ ^]^ The multistructure subcompartmentalized design of capsosomes (**Figure** **1**A) provides versatile drug‐loading capabilities, rendering capsosomes well suited for multidrug delivery. The liposome components favorably encapsulate small molecules, with hydrophilic molecules loaded within the lumen and hydrophobic compounds within the membrane.^[^ ^23^ ^]^

Here, we demonstrated the use of capsosomes as an advanced single‐ and dual‐antibiotic drug delivery system against MRSA infections in vitro and in vivo. The capsosomes were formulated to be responsive to bacterial toxins due to the composition of the contained lipidic compartments. Through incubation of the capsosomes with various MRSA strains, we showed that release of model cargoes was mediated by bacterial toxins, which are regulated by the *agr* locus. Treating the capsosomes with synthetic PSMs confirmed that these toxins are one of the species involved in the release mechanism. We further determined that the cargo release was specific to pathogenic bacteria by showing that a representative non‐pathogenic bacterium *Lactococcus lactis* did not cause any release. Subsequent tests with single‐ (vancomycin) and dual‐ (vancomycin and antimicrobial peptide) loaded capsosomes revealed successful bacteria‐activated killing of MRSA in vitro, whilst the dual configuration provided a synergistic effect. The bactericidal efficacy of capsosomes was further confirmed in an in vivo *Drosophila melanogaster* MRSA infection model. Thus, our capsosome‐based single‐ and dual‐antibiotic drug delivery system presents a flexible and versatile platform for the further development of localized bacteria‐activated multiantibiotic drug prophylaxis.

## 2 Results and Discussion

### 2.1 Assembly of Responsive Single‐ and Dual‐Loaded Multilayer Capsosomes

A multiantibiotic loaded microassembly was created with the aim of achieving a bacteria‐activated release of single and combinations of antibacterial compounds upon bacterial growth. For this purpose, we designed multilayer capsosomes, which were assembled in a hierarchical manner using a micron‐scale mesoporous silica core and exploiting electrostatic interactions between alternating oppositely charged layers of polymers and cargo‐loaded liposomes (Figure 1A). In contrast to other capsosome designs that use positively charged polymers and negatively charged or zwitterionic liposomes,^[^ ^21^, ^24^, ^25^ ^]^ we utilized a negatively charged polymer and positively charged liposomes. This strategy allowed deposition of positively charged liposomes as the final layer on the capsosomes, which could facilitate interaction with the negatively charged cell wall of Gram‐positive bacteria, hence maximizing the response speed and localization. The embedded liposomes within capsosomes serve as compartments for hydrophilic and hydrophobic antibiotics. Since the assembly of our system is based on a stepwise hierarchical assembly process, we first characterized the liposome component in isolation before incorporation into the capsosomes.

Cationic liposomes were formulated via thin‐film hydration, followed by extrusion. The liposome composition contained equimolar quantities of sphingomyelin and cholesterol, which were selected due to their known responsiveness towards bacterial toxins,^[^ ^26^, ^27^, ^28^, ^29^ ^]^ complemented with 25% (mol/mol) 1,2‐dioleoyl‐3‐trimethylammonium‐propane (DOTAP) to integrate the desired positive charge. Characterization of these liposomes with dynamic light scattering (DLS) revealed hydrodynamic diameters of 140 ± 59 nm (Figure S1A,B, Supporting Information). The zeta potential for these liposomes was measured as +45 ± 10 mV (Figure S1C, Supporting Information), confirming that the incorporated DOTAP indeed conferred an overall net positive surface charge to the liposomes.

Mesoporous silica microparticles (diameter 2.2 ± 0.2 µm, Figure S2A,B, Supporting Information) were selected as the core scaffold component since their micron‐scale size prevents rapid diffusion away from an injection or implantation site^[^ ^30^ ^]^ and there is some evidence of gradual degradation over 6–12 months.^[^ ^31^, ^32^ ^]^ Although not performed herein, the mesoporous silica could be removed after capsosome assembly but this involves usage of highly toxic HF.^[^ ^33^ ^]^ Incorporation of readily degradable polylactide core materials (e.g., PLGA) would represent an alternative option with a more defined biodegradation mechanism. To assemble the capsosomes, a cationic polymer, poly(allylamine hydrochloride) (PAH), was first adsorbed to the mesoporous silica. This layer of PAH was used to interface between the negatively charged mesoporous silica and the initial layer of an anionic polymer, poly(methacrylic acid) (PMA), in order to avoid adsorbing cationic liposomes directly to the silica, which could have disrupted the integrity of the liposomes.^[^ ^34^ ^]^ After deposition of this PAH layer, the first layer of PMA was used to facilitate electrostatic layer‐by‐layer (LbL) adsorption of the cationic liposomes. A modified PMA, functionalized with pyridine dithioethylamine (referred to as PMA‐PDA, Figure S3, Supporting Information), was used to allow for future crosslinking of the polymer layers, which could be beneficial for improving the stability of the capsosomes or introducing an additional stimuli‐responsive trigger.^[^ ^35^ ^]^ Following the adsorption of this PAH and PMA‐PDA dual layer onto the silica microparticles, alternating layers of cationic liposomes and PMA‐PDA polymer were added to yield a final capsosome assembly consisting of a total of one PAH, three PMA‐PDA and three liposome layers (Figure 1A).

The LbL assembly process was characterized by measuring the zeta potential after each deposition step (Figure 1B). The zeta potentials measured after consecutive application of each species to the anionic mesoporous silica microparticles indicated alternating surface charges, corresponding to the adsorbed species (Figure 1B). A fluorescent model lipophilic cargo compound (1,1‐dioctadecyl‐3,3,3,3‐tetramethylindodicarbocyanine, DiD, 1 wt%) was incorporated into the liposome membrane prior to capsosome formation to further study the assembly process and the loading of hydrophobic molecular cargo in our hierarchical system. To verify adsorption of the DiD‐loaded liposomes onto the capsosomes, changes in fluorescence intensity were measured throughout the assembly process using flow cytometry. The fluorescence intensities of capsosomes containing one, two, or three layers of DiD‐loaded liposomes increased according to the number of layers (Figure 1C; Figure S4, Supporting Information). Additionally, the magnitude of increase in fluorescence intensities was greater as layer number increased, representing the slightly larger surface area available for liposome binding. Nanoparticle tracking analysis (NTA) measurements of the concentration of remaining liposomes in the solutions after removal of the capsosomes allowed calculation of the efficiencies of liposome deposition (fraction immobilized): 78 ± 6% for layer one, 92 ± 12% for layer two, and 93 ± 36% for layer three (Figure 1D). These high deposition efficiencies are advantageous when considering application of our system utilizing expensive drug cargo.

To demonstrate the principle of assembling capsosomes carrying multiple drug cargoes with varied characteristics, we also loaded liposomes with a fluorescent hydrophilic model cargo (0.5 × 10^−3^ m sulforhodamine B, SRB). Capsosomes were then prepared containing two inner layers of SRB‐loaded liposomes to represent hydrophilic drug loading, and one outer layer of DiD‐loaded liposomes to represent hydrophobic drug loading. Fluorescence microscopy showed that both types of liposomes were successfully deposited on to the surface of the mesoporous silica core, although resolution limitations did not allow elucidation of the exact positions of individual layers (Figure 1E; Figure S5, Supporting Information). In conclusion, we demonstrated that capsosomes could be assembled in a LbL manner utilizing oppositely charged polymers and liposomes containing both hydrophilic and hydrophobic model cargoes.

### 2.2 Bacterial Toxin‐Triggered Release Mechanism from Liposomes within Capsosomes

Designing our capsosomes for antibiotic drug delivery applications, we have chosen a liposome formulation that is known to be responsive to bacterial toxins.^[^ ^14^, ^26^, ^27^, ^28^, ^29^ ^]^ Membrane‐damaging toxins are secreted virulence factors produced by many pathogenic bacteria to disrupt eukaryotic cell membranes for a burst release of nutrients. *S. aureus* produces a multitude of cytolytic toxins including *α*‐toxin, *β*‐toxin, and PSMs.^[^ ^17^ ^]^ We hypothesized that these secreted toxins could mediate drug release from our capsosomes.

**Figure 2:**
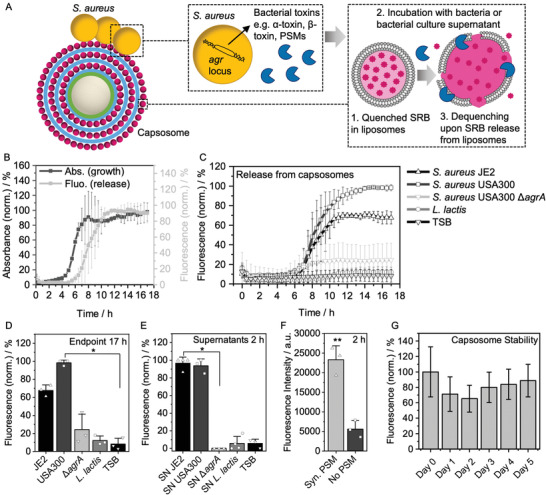
Toxin‐triggered release from capsosomes. A) Schematic depicting release of SRB from capsosomes triggered by *S. aureus* toxins, which are primarily controlled by the *agr* locus. B) Max–min normalized absorbance at 600 nm (indicating bacterial growth) and fluorescence intensity at 585 nm (indicating SRB release) of SRB‐capsosomes (initially containing a quenched concentration of SRB inside the liposomal compartments, 10 × 10^−3^ m) and *S. aureus* JE2 over 17 h (mean ± s.d., *N* = 3 independent experiments, data set in Figure S6, Supporting Information). C) Fluorescence intensity at 585 nm of SRB‐capsosomes incubated with *S. aureus* JE2 (from (B)), *S. aureus* USA300, *S. aureus* USA300 Δ*agr* mutant (low toxin expression), *L. lactis* and TSB over 17 h (mean ± s.d., *N* = 3 independent experiments, data set in Figure S6, Supporting Information). D) Comparison of endpoint fluorescence (17 h) from (C) (mean ± s.d.). Statistical significance was calculated using a Kruskal–Wallis ANOVA with a post hoc Dunn's test. *p* ≤ 0.05 (*). E) Max–min normalized fluorescence intensity after addition of bacterial supernatants to SRB‐capsosomes (mean ± s.d., *N* ≥ 3 independent experiments, data set in Figure S7, Supporting Information). Statistical significance was calculated using a Kruskal–Wallis ANOVA with a post hoc Dunn's test. *p* ≤ 0.05 (*). F) Fluorescence intensity after addition of synthetic PSMs to SRB‐capsosomes (mean ± s.d., *N* = 3 independent experiments, data set in Figure S8, Supporting Information). Statistical significance was calculated using an unpaired, two‐tailed Student's t‐test. *p* ≤ 0.01 (**). G) Flow cytometry analysis of SRB‐capsosomes stability (initially containing a quenched concentration of SRB inside the liposomal compartments, 10 × 10^−3^ m) after incubation in TSB at 37 °C (median ± s.d, *N* = 1, particles measured per time point ≥ 19 000).

To study this toxin‐triggered release mechanism, we prepared capsosomes containing three layers of SRB‐loaded liposomes at a self‐quenching concentration (10 × 10^−3^ m,^[^ ^36^ ^]^ henceforth referred to as SRB‐capsosomes). Toxin activity was expected to cause release of the SRB and an accompanied dequenching that could be detected as an increase in fluorescence (**Figure** **2**A). SRB‐capsosomes were incubated with *S. aureus* JE2, a highly virulent MRSA strain,^[^ ^37^ ^]^ which is known to upregulate toxin expression during late exponential/early stationary growth phase due to activation of the Agr quorum‐sensing system.^[^ ^38^, ^39^ ^]^ A sharp increase in fluorescence intensity was observed in the sample as *S. aureus* entered the stationary phase after ≈7.5 h, which is in agreement with the expected time of cytolytic toxin production (Figure 2B; Figure S6A, Supporting Information). A direct correlation was noted between the increase in fluorescence intensity and an observed increase in optical density, attributed to an increase in turbidity due to bacterial growth (Figure 2B; Figure S6A, Supporting Information). It was therefore confirmed that, as the bacterial culture grew, toxins were produced, facilitating lysis of the liposomes and concomitant release of model hydrophilic cargo (SRB).

To test that secreted toxins were responsible for cargo release we used wild type *S. aureus* JE2 and *S. aureus* USA300, LAC strains^[^ ^37^ ^]^ and a mutant of the USA300, LAC strain,^[^ ^40^ ^]^ which lacked the *agrA* gene (*S. aureus ΔagrA*) an essential gene for triggering cytoloytic toxin production.^[^ ^41^ ^]^ As expected, incubation of the capsosomes with *S. aureus* JE2 and *S. aureus* USA300 LAC wild type strains resulted in a large increase in fluorescence intensity over the 17 h incubation time (Figure 2C,D; Figure S6B, Supporting Information). In contrast, capsosomes incubated with *S. aureus ΔagrA* showed negligible increase in fluorescence intensity after 17 h incubation, indicating that the low level of toxin production in this mutant strain was not sufficient to trigger release of SRB from the capsosomes compared to the *S. aureus* USA300 strain (Figure 2C,D; Figure S6B, Supporting Information). Furthermore, a similar increase in absorbance was observed for *S. aureus* JE2, *S. aureus* USA300 LAC, and *S. aureus ΔagrA*, confirming that all strains grew similarly and hence the differences in SRB release can be attributed to differing levels of toxin production (Figure S6C, Supporting Information).

Next SRB‐capsosomes were also incubated with *L. lactis*, which is a non‐pathogenic and non‐toxin producing bacterial strain, to further test the hypothesis that bacterial toxins were responsible for hydrophilic cargo release from the liposomes within capsosomes. Growth of *L. lactis* was observed as an increase in absorbance at 600 nm at ≈9 h (Figure S6D, Supporting Information), but this growth was not accompanied by an increase in fluorescence intensity of the SRB (Figure 2C,D; Figure S6E, Supporting Information). Therefore, this data provided further evidence that toxins produced by pathogenic bacteria facilitated the release of hydrophilic model cargo (SRB), while the capsosomes stayed intact in the medium alone (TSB, Figure 2C,D) as also measured by flow cytometry (dye remained quenched, Figure 2G). Due to the use of quenched concentration of SRB (10 × 10^−3^ m), the initial drop in relative fluorescence is attributed to further quenching (not release), while the median fluorescence never reached above the initial fluorescence (day 0), confirming retention of high, quenched SRB concentrations over at least 5 days and hence demonstrating high stability of our system. To further confirm that the release mechanism was due to soluble secreted toxins, we repeated these experiments by mixing sterile filtered bacteria culture supernatants with SRB‐capsosomes rather than incubating them directly with the bacteria. This supernatant data reproduced the bacteria data (Figure 2E; Figure S7, Supporting Information), which further supported a toxin‐induced release mechanism. Additionally, direct addition of synthetic PSMs to SRB‐capsosomes led to a much higher fluorescence intensity compared to the SRB‐capsosomes alone (Figure 2F; Figure S8, Supporting Information), supporting the involvement of PSMs in the lytic drug release process.

Overall, these findings strongly support the hypothesis of a bacterial toxin‐triggered release of hydrophilic molecules from liposomes contained within the capsosomes. This triggerable release mechanism could be highly beneficial for selectively releasing antibiotics in vivo only when pathogenic toxin‐producing bacteria are present, mitigating the negative impact of antibiotics on the commensal microbiota.

### 2.3 Antibacterial Activity of Capsosomes Loaded with Vancomycin

After successful demonstration of hydrophilic cargo loading and bacteria toxin‐triggered release from capsosomes, we loaded the capsosomes with vancomycin, a hydrophilic glycopeptide which inhibits cell‐wall synthesis,^[^ ^42^ ^]^ because it is an important frontline antibiotic for the treatment of infections caused by MRSA.^[^ ^43^, ^44^ ^]^ However, common adverse effects of vancomycin treatment include nephrotoxicity and ototoxicity, highlighting the need for improved antibiotic delivery.^[^ ^45^ ^]^

**Figure 3:**
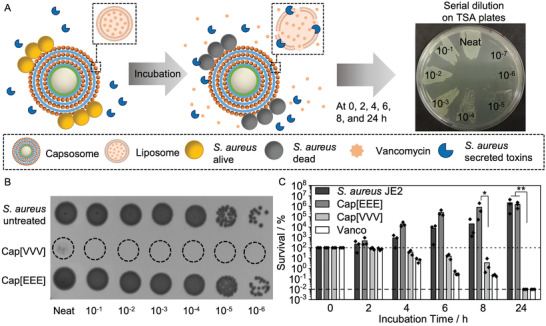
Antibacterial activity of vancomycin‐loaded capsosomes. A) Schematic depicting killing assay of vancomycin‐loaded capsosomes (Cap[VVV]) with *S. aureus* JE2. B) A spot‐on assay showing *S. aureus* JE2 untreated and incubated with Cap[VVV] and empty capsosomes (Cap[EEE]) after 24 h (prepared for visual comparison of the antibacterial effect of the capsosomes). C) Antibacterial activity of Cap[VVV], Cap[EEE], and free vancomycin (concentration 4 µg mL^–1^) against *S. aureus* JE2 (*N* = 3 independent experiments, *n* = 1 technical repeat). Bars show mean values. Dashed line indicates limit of detection with no colony forming units detectable and dotted line indicates 100% survival. Note: survival was counted from plated serial dilutions as depicted in (A) and not the displayed spot‐on assays in (B). Cap[VVV]: Three layers of vancomycin loaded liposomes, Cap[EEE]: Three layers of empty liposomes. Note: 5 mg of capsosomes were incubated with bacteria. Statistical significance was calculated using a Kruskal–Wallis ANOVA with a post hoc Dunn's test (comparisons to Cap[VVV]). *p* ≤ 0.05 (*), *p* ≤ 0.01 (**).

Similarly to the SRB‐loaded capsosomes, capsosomes containing three layers of vancomycin‐loaded liposomes were prepared (henceforth referred to as Cap[VVV]). First, vancomycin loading into liposomes was quantified using analytical HPLC, and found to be 6.5 ± 1.6 µg mL^–1^ per 1 mg mL^–1^ liposomes, with a loading efficiency of 2.9 ± 0.7% (Figure S9, Supporting Information). Cap[VVV] contained a maximum of 19.5 µg vancomycin per 5 mg capsosomes assuming all the liposomes were loaded (actual value will be lower due to some loss during preparation, see measurement of binding capacity) (Figure 1D). To assess antibacterial effect of Cap[VVV], a killing assay was performed, in which the clinically relevant MRSA strain (*S. aureus* JE2)^[^ ^46^ ^]^ was incubated with the capsosomes and the survival measured (**Figure** **3**A).

A strong killing effect was observed after incubation of 5 mg Cap[VVV] with *S. aureus* JE2, with >99.99% of bacteria were killed after 24 h (Figure 3B,C; Figure S10, Supporting Information). Together with the SRB‐capsosome data from above and the hydrophilic nature of both cargoes (SRB and vancomycin), this data suggests successful toxin‐mediated vancomycin release from Cap[VVV]. Empty capsosomes (Cap[EEE]) exhibited no killing effect, indicating the Cap[VVV] killing effect was mediated by the release of vancomycin. As expected, treatment with free vancomycin was also highly effective. We did not intend to improve activity of vancomycin within this project but rather design a controlled release system that could be used in a localized and pathogenic bacteria growth‐activated manner. A previous study reported bacterial toxin‐triggered release of vancomycin from gold nanoparticle‐stabilized liposomes,^[^ ^12^ ^]^ however, the advantage of our hierarchical capsosome system is the capacity to anchor large numbers of nanoscale liposomes in the proximity of a bacterial infection due to the microscale size of the capsosomes, which allows localized infection control.

### 2.4 Synergistic Antibacterial Activity of Dual‐Loaded Capsosomes with Vancomycin and Antibacterial Peptide

Combination antibiotic therapies, which involve the coadministration of multiple antibacterial compounds, are increasingly employed clinically to exploit synergistic relationships between antibiotics, to reduce the chance of establishment of antibiotic resistance, and to broaden the antibacterial spectrum for unknown or polymicrobial infections.^[^ ^47^, ^48^ ^]^ The clinical use of vancomycin in combination with other antibiotics is important because vancomycin alone lacks efficacy, failing in around 20–50% of infections.^[^ ^49^, ^50^, ^51^ ^]^ Furthermore, vancomycin treatment can lead to the emergence of strains with reduced susceptibility (vancomycin intermediate *S. aureus*, VISA). As such, it is hoped that combination of vancomycin with another antimicrobial might improve efficacy and reduce the emergence of VISA strains.

Since our capsosomes offer the possibility of loading and colocalizing both hydrophilic and hydrophobic compounds (Figure 1A), we screened a wide variety of antibacterial compounds with different physicochemical characteristics for synergistic killing effects with our previously selected antibiotic, vancomycin (Figure S11, Supporting Information). Combination of vancomycin and a recently developed antibacterial peptide, Palm‐Arg‐Arg‐NH_2_,^[^ ^52^ ^]^ synthesized by solid‐phase synthesis (Figure S12, Supporting Information), provided a 1.8 × 10^2^ fold and 1.5 × 10^4^ fold higher killing against *S. aureus* JE2, compared to vancomycin or Palm‐Arg‐Arg‐NH_2_ alone, respectively (Figure S11A, Supporting Information). With a >2 log_10_ difference between the combined and individual drug effects, this drug combination was deemed synergistic.^[^ ^53^ ^]^ Palm‐Arg‐Arg‐NH_2_ is believed to function by disrupting the bacterial cell membrane due to its structure containing a lipophilic palmitic acid component, which facilitates insertion into lipid membranes, and two arginine residues to provide a strong positive charge.^[^ ^52^ ^]^ Membrane disrupting antimicrobial peptides are thought to be less prone to resistance development, making them an attractive class of antibacterial compounds.^[^ ^54^ ^]^ However, clinical implementation of antimicrobial peptides has been limited due to their cytotoxic effect towards human cells.^[^ ^55^ ^]^ We performed a hemolysis assay as a measure of cell toxicity and determined that Palm‐Arg‐Arg‐NH_2_ displayed a hemolytic effect above a concentration of 64 µg mL^1^ (Figure S13, Supporting Information), which was used as a boundary for loading of the capsosomes.

**Figure 4:**
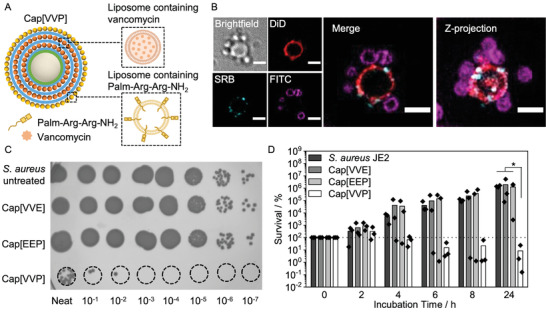
Synergistic antibacterial activity of dual‐loaded capsosomes. A) Schematic depicting design of dual‐loaded capsosomes containing two inner layers of vancomycin loaded liposomes and one outer layer of Palm‐Arg‐Arg‐NH_2_‐loaded liposomes. B) Widefield deconvolution fluorescence microscopy showing dual‐loaded capsosome (model dye system from Figure 1E), incubated with FITC‐labeled *S. aureus* JE2. Scale bars: 2 µm. More images shown in Figure S15, Supporting Information. C) Spot‐on assay showing *S. aureus* JE2 untreated, and incubated with Cap[VVP], Cap[VVE], and Cap[EEP]. D) Antibacterial activity of capsosomes containing only vancomycin (Cap[VVE]), capsosomes containing only Palm‐Arg‐Arg‐NH2 (Cap[EEP]), and capsosomes containing both vancomycin and Palm‐Arg‐Arg‐NH_2_ (Cap[VVP]) against *S. aureus* JE2 (*N* = 3 independent samples, *n* = 1 technical repeat). Bars show mean values. Cap[VVP]: Layer one and two are vancomycin loaded liposomes, layer three are antibacterial peptide loaded liposomes. Cap[VVE]: Layer one and two are vancomycin loaded liposomes, layer three are empty liposomes. Cap[EEP]: Layer one and two are empty liposomes, layer three are antibacterial peptide loaded liposomes. Note: In comparison to Figure 3 only 1.25 mg capsosomes were incubated with bacteria to reach a concentration of Cap[VVE] and Cap[EEP] which does not show antibacterial activity to evaluate potential synergies for Cap[VVP]. Dotted line indicates 100% survival. Statistical significance was calculated using a Kruskal–Wallis ANOVA with a post hoc Dunn's test (comparisons to Cap[VVP]). *p* ≤ 0.05 (*).

Analogous to the loading of the two model compounds above (SRB and DiD, Figure 1E; Figure S5, Supporting Information), we incorporated vancomycin and Palm‐Arg‐Arg‐NH_2_ in our capsosome system (**Figure** **4**A). To assemble these dual‐antibiotic loaded capsosomes, vancomycin and Palm‐Arg‐Arg‐NH_2_ were first loaded into separately prepared liposomes (see vancomycin loading and liposome composition information above). Palm‐Arg‐Arg‐NH_2_ incorporation into liposomes was assessed with quantitative mass spectrometric analysis, revealing loading of 13.6 ± 1.2 µg mL^–1^ per 1 mg mL^–1^ liposomes and a loading efficiency of 20 ± 2% (Figure S14, Supporting Information). Following liposome loading, capsosomes were prepared containing two inner layers of vancomycin‐loaded liposomes and one outer layer of liposomes containing Palm‐Arg‐Arg‐NH_2_ (henceforth referred to as Cap[VVP]) (Figure 4A). Dual loaded Cap[VVP] contained a maximum of 13 µg vancomycin and 13.6 µg peptide per 5 mg capsosomes. Before studying the antibacterial effect of Cap[VVP], we first visualized the interaction of dual‐loaded capsosomes and *S. aureus* JE2 (labeled with FITC) by employing the SRB‐DiD capsosomes from above (Figure 1E). The bacteria were found to form an interaction with the surface of the capsosomes, indicating that the positively charged capsosome exterior could potentially improve the antibacterial activity by bringing bacteria, which have a negative surface charge, into proximity of the drugs released from the capsosomes (Figure 4B; Figure S15, Supporting Information).

To assess the antibacterial killing effect of dual‐loaded capsosomes (Cap[VVP]), different dilutions of capsosomes (2.5, 1.25 and 0.625 and 0.312 mg) were then incubated with *S. aureus* JE2 for 24 h at 37 °C, after confirming that these capsosome concentrations did not result in hemolytic toxicity (Figure S16, Supporting Information). Note, 1.25 mg capsosomes comprising two layers of vancomycin‐loaded liposomes and one layer of empty liposomes (Cap[VVE]) are now containing a lower concentration of vancomycin than the 5 mg of Cap[VVV] utilized in Figure 3. Here, incubations at lower capsosome concentrations compared to Figure 3 were conducted in order to resolve the differences in activity between single‐ and dual‐loaded capsosomes. Incubation of 1.25 mg Cap[VVP] with *S. aureus* JE2 resulted in 91% killing after 24 h, indicating a bactericidal effect (Figure 4C,D). Conversely, *S. aureus* JE2 incubated with the single drug controls at the same capsosome concentration (Cap[VVE] and Cap[EEP]) exhibited no appreciable growth deterrence, demonstrating the benefit of coloading a synergistically active drug mixture within capsosomes. We envision that our capsosome system offers the advantages of colocalizing delivery of the two synergistic antibacterial compounds to a particular site in the body and facilitating release of the compounds only in the event of infection by a toxin‐producing strain of bacteria.

### 2.5 In Vivo Activity of Antibiotic‐Loaded Capsosomes

**Figure 5:**
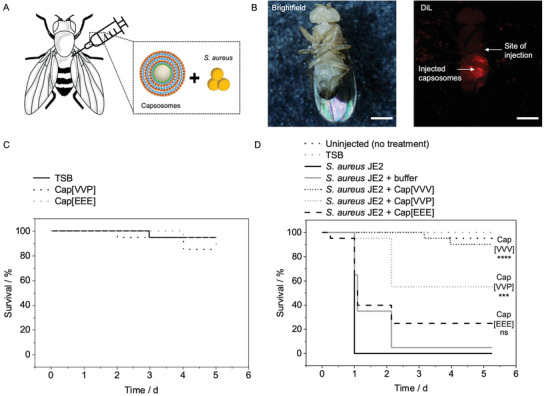
In vivo antibacterial activity of capsosomes. A) Schematic depicting intra‐abdominal coinjection of *D. melanogaster* (fruit fly) with capsosomes and *S. aureus* JE2. B) Brightfield and fluorescence micrographs of DiL‐capsosomes (without bacteria) injected into the abdomen of flies. Scale bars: 200 µm. C) Survival of flies injected with Cap[VVP] and Cap[EEE] without bacteria. D) Survival of flies infected with *S. aureus* JE2 and treated with Cap[VVV], Cap[VVP], and Cap[EEE], or buffer control. Additionally, control groups of flies were left uninjected or uninfected (shown are representative, *N* = 1 independent experiment, *n* = 20 flies, repetition shown in Figure S17, Supporting Information). Statistical significance was calculated using the pairwise_survdiff function in R, which carries out pairwise comparisons using the Log‐Rank test, with a Bonferroni *p* value adjustment method. *p* ≤ 0.0001 (****), *p* ≤ 0.001 (***), ns = non‐significance. *p* values shown are comparing *S. aureus* JE2 + Cap[VVV] or Cap[VVP] to *S. aureus* JE2 + buffer. Cap[VVV]: Three layers of vancomycin loaded liposomes. Cap[VVP]: Layer one and two are vancomycin loaded liposomes, layer three are antibacterial peptide loaded liposomes. Cap[EEE]: Three layers of empty liposomes. Schematic in A modified from Servier Medical Art website CC‐BY.

Following determination of the in vitro killing activity of Cap[VVV] and Cap[VVP] against *S. aureus* JE2, the in vivo efficacy of these capsosomes was studied using *Drosophila melanogaster* (fruit fly) as a model system for MRSA infection (**Figure** **5**A). *D. melanogaster* is a well‐established model system for studying infection and immune responses.^[^ ^56^, ^57^ ^]^ Infection of *D. melanogaster* with *S. aureus* has previously been studied and found to cause systemic infection and rapid death of the flies within 24 h.^[^ ^58^ ^]^ Furthermore, bactericidal activity of antibiotic‐loaded nanomaterials (e.g., tigecycline in chitosan‐based nanogels) has been successfully studied in the past using a *D. melanogaster* model infected with *S. aureus*, utilizing fly survival, which negatively correlated with the number of bacteria recovered from the flies, as a readout for antibacterial activity.^[^ ^59^ ^]^ In contrast to vertebrates, flies have an open circulatory system, where hemolymph is in direct contact with the fly organs, hence any injected material potentially distributes widely. Between samples, some variation in distribution is expected, but keeping the injection volume identical (50 nL) for all the flies and using large experimental groups (*n* = 20 flies each) helps to minimize the effect of some variability in injections.

The suitability of *D. melanogaster* as a model system for testing the antibacterial activity of the capsosomes was first confirmed by injecting DiL‐capsosomes into the abdomen of the flies, with observation of dotted DiL fluorescence within the flies verifying that the capsosomes could be successfully injected (Figure 5B; Figure S17A, Supporting Information). Then, we tested whether fly survival is dependent on the bacterial load (*S. aureus* JE2), which revealed that higher bacteria numbers caused more rapid fly death, confirming the suitability of fly survival as a readout for antibacterial activity (Figure S17B, Supporting Information). Next, the flies were injected with Cap[EEE], Cap[VVP], or TSB as a control to assess whether the capsosomes alone were toxic to flies. After 5 d, there was no statistically significant difference in the survival of the flies injected with the capsosomes compared to the TSB control, indicating that the capsosomes were compatible with the flies (Figure 5C).

To assess the antibacterial activity of capsosomes, the flies were injected with a mixture of *S. aureus* JE2 and either Cap[VVV], Cap[VVP], Cap[EEE], or a buffer control. This in vivo process was carried out at the higher capsosome concentration of the in vitro assay for Cap[VVV] (Figure 3) and loading all the three layers with antibiotics to maximize the desired protective effect. Capsosomes and *S. aureus* JE2 were mixed to yield a suspension containing the same amount of capsosomes for all samples (5 mg silica) and ≈2 × 10^4^ CFU mL^–1^ bacteria, followed by coinjection of 50 nL of this mixture into the flies. A coinjection model (Figure 5A) was selected for the in vivo assay to mimic a scenario in which bacterial contamination could be introduced to the body upon surgical implantation of a medical device, during which capsosomes could also be applied to the site. Control samples were also prepared in which the flies were not injected with anything or were injected only with TSB media. After 1 d, 95–100% of the flies infected with *S. aureus* JE2 had died, indicating the lethality of the bacteria applied to the flies (Figure 5D; Figure S17, Supporting Information). In contrast, 50–90% of the flies injected with Cap[VVV] survived after 5 d, which was a statistically significant increase in survival compared to the flies injected with *S. aureus* JE2 and buffer. This finding suggested that vancomycin released from Cap[VVV] led to greater fly survival by reducing the bacterial load, providing proof of concept that this approach has prophylactic value.

Survival of the flies injected with the dual‐loaded capsosomes (Cap[VVP]) was 15–55% after 5 d, which was significantly greater than the flies injected with *S. aureus* JE2 and buffer, but only in one of the independent experimental repeats. Therefore, Cap[VVP] was found to be less effective than Cap[VVV] in an in vivo environment. A potential reason for Cap[VVV] being more effective than Cap[VVP] in the fly model could be that the peptide (Palm‐Arg‐Arg‐NH_2_) may be less potent in more complex in vivo environments due to the presence of competing hydrophobic environments such as host cell membranes, which can hinder distribution of the peptide. Especially with respect to bacteria that ended up far away from the capsosomes after injection (due to the hemolymph), it is expected that released vancomycin is more likely to reach these bacteria compared to the amphiphilic peptide, hence, limiting the overall in vivo activity of the peptide. In addition, the effective available vancomycin concentration was higher for Cap[VVV] compared to Cap[VVP], because the in vivo data was conducted under conditions to maximize an effect, filling the last layer of Cap[VVE] (Figure 4) with vancomycin too. Nonetheless, the dual‐loaded Cap[VVP] system could still offer possible advantages over the Cap[VVV] system since the drug combination could aid in preventing the development of antibiotic resistance. Hence, optimization of the Palm‐Arg‐Arg‐NH_2_ concentration and/or the number of liposome layers could potentially improve the efficacy of the Cap[VVP] system. Overall, this in vivo demonstration of single‐ and dual‐antibiotic delivery from capsosomes provides the fundamental basis for their potential future use for localized and pathogenic bacteria triggered antibiotic drug delivery.

## 3 Conclusion

We showed that hierarchical multilayer capsosomes composed of a mesoporous silica microparticle core, followed by alternating layers of oppositely charged polymers and liposomes, could function as single‐ and dual‐antibiotic drug reservoirs that respond to bacterial growth. We demonstrated MRSA bacterial toxin‐triggered release from the capsosomes, providing evidence that toxins under control of the *agr* locus are largely responsible for this release mechanism from liposomes containing sphingomyelin and cholesterol. We then loaded these liposomes with the clinically relevant antibiotic, vancomycin, and demonstrated that capsosomes containing three layers of vancomycin‐loaded liposomes (Cap[VVV]), displayed >99.99% killing of an MRSA strain, *S. aureus* JE2. We further determined that vancomycin had synergistic activity in vitro in combination with an antibacterial peptide, Palm‐Arg‐Arg‐NH_2_, and exploited this drug relationship within the capsosomes, which were also found to provide a synergistic effect in vitro when loaded with both compounds. Finally, we further demonstrated that prevention of MRSA infection in *D. melanogaster* with antibiotic‐loaded capsosomes led to a statistically significantly improved survival over 5 d, compared to control organisms treated with buffer, illustrating that the capsosomes maintained their antibacterial activity in a complex in vivo environment. We found that, even in this challenging in vivo environment, the Cap[VVV] functioned successfully and protected the host flies from the bacterial infection. Interestingly the Cap[VVV] construct performed better than the dual‐loaded Cap[VVP] construct, although the dual‐loaded capsosomes could still provide additional benefits for reducing the likelihood of resistance development, which will have to be tested further in the future. We envision that the single‐ and dual‐loaded capsosomes developed here could be applied to treat or prevent infection of long‐term medical device implants. Use of these capsosomes could prevent the need for systemic application of high concentrations of antibiotics, reducing the risk of side effects and the development of antibiotic resistance. Although thorough in vivo investigations are needed to evaluate whether nephrotoxicity can be reduced through a localized and controlled release from our capsosomes, they provide an optimal carrier for multiple drugs with various solubilities (hydrophobic and hydrophilic) and beyond vancomycin. Further, the bacterial toxin‐triggered release mechanism will ensure that delivery of the antibiotics occurs only at the time and site of an infection. Overall, we have demonstrated engineering of an advanced hierarchical microassembly (capsosomes) for controlled delivery of the clinically relevant antibiotic, vancomycin, and combination delivery of vancomycin and an antimicrobial peptide (Palm‐Arg‐Arg‐NH_2_) both in vitro and in vivo. These constructs demonstrate the application of multipurpose capsosomes towards antibiotic drug delivery, providing a platform for new single‐ and multiantibiotic precision drug delivery strategies and their advancement towards clinical applicability.

## 4 Experimental Section

### Materials

Cholesterol, sulforhodamine B (SRB), palmitic acid, mesoporous silica microparticles (2 µm diameter, 4 nm pore diameter), cysteamine hydrochloride, 2,2’‐dipyridyl disulfide, poly(allylamine hydrochloride) (≈65 kDa, 10 wt% in H_2_O) (PAH), tricine, sodium chloride, piperidine, diethyl ether (DEE), trifluoracetic acid (TFA), Triton X‐100, fluorescein isothiocyanate (FITC) isomer I, M17 agar, glucose and sucrose were purchased from Sigma‐Aldrich, UK. Fmoc‐L‐Arg(Pbf)‐OH, 1‐ethyl‐3‐(3‐dimethylaminopropyl)carbodiimide hydrochloride (EDC‐HCl), and (1‐[Bis(dimethylamino)methylene]‐1*H*‐1,2,3‐triazolo[4,5‐b]pyridinium 3‐oxide hexafluoro‐phosphate (HATU) were purchased from Fluorochem, UK. Brain sphingomyelin and 1,2‐dioleoyl‐3‐trimethylammonium‐propane (DOTAP) were purchased from Avanti Polar Lipids, USA. Poly(methacrylic acid) (≈15 kDa, 30 wt% solution in H_2_O) was purchased from Polysciences, USA. Rink Amide AM resin (100–200 mesh, 0.4–0.75 mmol g^–1^) was purchased from Iris Biotech, Germany. Diisopropylethylamine (DIPEA) was purchased from AGTC Bioproducts. 1,1″‐dioctadecyl‐3,3,3″,3'‐tetramethylindodicarbocyanine perchlorate (DiD) and Dulbecco's phosphate buffered saline (PBS) without phenol red, calcium, and magnesium (Gibco) were purchased from Thermo Fisher Scientific, UK. Vancomycin was purchased from PanReac AppliChem, Germany. *N*,*N*‐dimethyl formamide (DMF), water (HPLC grade) and methanol were purchased from VWR chemicals, UK. Dichloromethane (DCM), triisopropylsilane, and acetonitrile (HPLC grade) were purchased from ACROS Organics, USA. Sheep blood (defibrinated) was purchased from Southern Group Laboratory Ltd, UK. Tryptic soy agar (TSA) and tryptic soy broth (TSB) were purchased from BD Biosciences, USA. Paraformaldehyde (16% aqueous solution) was purchased from Electron Microscopy Sciences, USA. Synthetic PSMs were purchased from Peptide Protein Research Ltd, UK. All chemicals were used as received unless otherwise indicated.

### Poly(methacrylic acid) Functionalized with Pyridine Dithioethylamine (PMA‐PDA)

Pyridine dithioethylamine (PDA) was synthesized as described previously.^[^ ^60^ ^]^ Briefly, 2.45 g of cysteamine hydrochloride in 10 mL methanol was added dropwise to 150 mL methanol containing 25 g of 2,2’‐dipyridyl disulfide over 30 min. The reaction mixture was then stirred continuously under an atmosphere of argon for 24 h, after which the product was isolated by precipitation using DEE (cooled to −20 °C). The synthesized compound was verified using LCMS and proton nuclear magnetic resonance spectroscopy (^1^H NMR in DMSO‐d6). The PDA was then lyophilized and stored at −80 °C.

To functionalize PMA with PDA, a solution containing 3.23 mL of 20 mg mL^–1^ 1‐ethyl‐3‐(3‐dimethylaminopropyl)carbodiimide hydrochloride (EDC‐HCl) in phosphate buffer and 300 mg of 30 wt% PMA solution (15 kDa) was prepared and stirred for 15 min (stir speed of 2). Next, either 0.5, 1.0, 1.5, or 2.5 mL of 20 mg mL^–1^ PDA in dH_2_O was added to the solution, followed by a 16 h incubation under stirring. The product was then dialyzed using Spectra/Por 3 dialysis tubing (3.5 kDa MWCO, 45 mm, Spectrum Laboratories, USA) in dH_2_O over 1.5 d, replacing the dH_2_O four times, and then lyophilized. The degree of functionalization was determined using ^1^H NMR.

### Synthesis of Palm‐Arg‐Arg‐NH_2_

Palm‐Arg‐Arg‐NH_2_ was synthesized using a standard Fmoc solid‐phase synthesis protocol.^[^ ^61^ ^]^ A 20 mL syringe equipped with a frit was filled with 1 mmol Rink Amide AM resin (100–200 mesh, 0.4–0.75 mmol g^–1^) and swelled in 20 mL dichloromethane (DCM) for 15 min. Fmoc deprotection was completed by incubating the resin twice in 20 mL of 20 vol% piperidine in *N,N*‐dimethyl formamide (DMF) for 15 min, and then washing the resin three times in 20 mL DMF, once in 20 mL methanol, and once in 20 mL DCM. Fmoc‐L‐Arg(Pbf)‐OH and palmitic acid were coupled to the resin using 3× molar equivalents with 3× molar equivalents of (1‐[Bis(dimethylamino)methylene]‐1*H*‐1,2,3‐triazolo[4,5‐b]pyridinium 3‐oxide hexafluoro‐phosphate (HATU) dissolved in a total of 12 mL DMF in the presence of diisopropylethylamine (DIPEA, 5 equivalents), to activate the amino acid and fatty acid. The activated amino acid or fatty acid solution was incubated with the resin on an orbital shaker for 1 h (Arg coupling) or overnight (palmitic acid coupling) and the resin was then washed as described above. Solvents were removed using a vacuum pump and ninhydrin tests (Cambridge Biosciences, UK) were completed to verify the presence or absence of free amine moieties after Fmoc deprotection step and amino acid coupling. Following synthesis, the resin was incubated in a round bottomed flaskunder stirring for 4 h in 50 mL of 95 vol% trifluoroacetic acid (TFA), 2.5 vol% triisopropylsilane, 2.5 vol% dH_2_O to cleave the peptide from the resin. The resin was then filtered through a frit and washed once with 20 mL TFA to collect the cleaved peptide, followed by removal of the TFA under reduced pressure. The peptide was recovered by precipitation using three washes with 100 mL diethyl ether (DEE), removing the DEE by centrifugation (5000 × g, 10 min, 4 °C).

The peptides were dissolved in 70 vol% dH_2_O, 30 vol% acetonitrile with 0.1 vol% TFA and filtered using a 0.45 µm filter (Corning, USA). Purification was carried out using reverse‐phase preparative high‐performance liquid chromatography (Shimadzu, Japan) on a Phenomenex C18 Gemini NX column (5 mm particle diameter, 110 Å pore diameter, 150 x 21.2 mm). Samples were run at 10 mL min^–1^ with the following 30 min protocol: 95% A (dH_2_O, 0.1 vol% TFA) and 5% B (acetonitrile, 0.1 vol% TFA) for5 min, a gradient from 5% to 95% B in 20 min, maintenance of 5% A 95% B for 5 min. Absorbance was measured at 220 nm. The peptide mass was verified using LCMS. The peptide was then lyophilized and stored at −20 °C.

### Scanning Electron Microscopy

A 50 µL aliquot of mesoporous silica microparticles (806 951, Sigma Aldrich, UK) suspended in 100% ethanol was applied to a glass slide. Samples were sputter‐coated with gold for 30 s at 20 mA. Images were taken using a JEOL JSM‐5610LV microscope with an operation voltage of 20 kV. Microparticle diameters were measured from SEM micrographs using FIJI.^[^ ^62^ ^]^

### Preparation of Liposomes

Liposome compositions were 37.5 mol% sphingomyelin, 37.5 mol% cholesterol, 25 mol% DOTAP, with 0.2 or 1 wt% DiD if included. Lipid films were prepared by applying 12 mg total lipids to 2 mL glass vials and removing the chloroform by evaporation using N_2_ gas, followed by desiccation for overnight. Lipid films were then hydrated with the desired buffer and cargo and incubated on a vortex mixer for 30 min at 1200 rpm and then subjected to five freeze‐thaw cycles, heating to 70 °C and freezing at –80 °C. The lipid suspensions were then extruded to 100 nm (10× through a 200 nm membrane and then 21× through a 100 nm membrane).

Empty liposomes were hydrated in 1.2 mL tricine buffer (buffer composed of 50 × 10^−3^ m tricine, 50 × 10^−3^ m NaCl, pH 7.5). Liposomes containing SRB were hydrated in 1.2 mL of 10 × 10^−3^ m SRB in tricine buffer. Liposomes loaded with vancomycin were hydrated in 0.6 mL of 4 mg mL^–1^ vancomycin in tricine buffer. Liposomes containing Palm‐Arg‐Arg‐NH_2_ were prepared by adding 500 µg Palm‐Arg‐Arg‐NH_2_ in 100 µL methanol during lipid film deposition onto the glass vial and then hydrating in 1.2 mL tricine buffer.

Non‐incorporated SRB, vancomycin, and Palm‐Arg‐Arg‐NH_2_ was removed from liposome suspensions using PD MiniTrap G‐25 (VWR, UK) and PD MidiTrap G‐25 (VWR, UK) columns, according to the provided gravity protocol. To ensure complete separation of liposomes from drug, liposomes loaded with vancomycin were diluted 4× prior to purification. Liposomes loaded with SRB and Palm‐Arg‐Arg‐NH_2_ were purified undiluted. 1× PD MiniTrap G‐25 (VWR, UK) and 2× PD MidiTrap G‐25 (VWR, UK) columns were used sequentially to yield high purity.

Liposomes were characterized by measuring dynamic light scattering (DLS) and zeta potential using a Zetasizer Nano ZS (Malvern, UK) with a scattering angle of 173° and a laser beam wavelength of 633 nm. For DLS, samples were diluted (10× dilution of 1 mg mL^–1^ lipid content, or equivalent) and measured three times in disposable micro cuvettes, each measurement comprising 15 runs. The measurements were then averaged. The zeta potential was measured by applying 50 µL of liposomes (1 mg mL^–1^ lipid content) and 950 µL 300 × 10^−3^ m sucrose to a folded capillary zeta cell. Three measurements (10–15 runs each) were taken and averaged. Liposome concentrations for capsosome assembly were determined using nanoparticle tracking analysis (NTA), see below.

Loading of vancomycin and Palm‐Arg‐Arg‐NH_2_ into liposomes was assessed using analytical HPLC and LCMS, respectively. Samples were prepared for analysis by mixing equal volumes of methanol and liposome sample for 10 min to lyse the liposomes and then filtering using a 0.2 µm PFTE filter (Phenomenex, USA).

### Assembly of Capsosomes

Generally, samples containing 5 mg mesoporous silica microparticles were mixed with 1 mL of 1 mg mL^–1^ poly(allylamine hydrochloride) (PAH) in dH_2_O in an AccuTherm microtube shaking incubator (Labnet, USA) at 400 rpm for 30 min. The PAH was removed using centrifugation (3000 × g, 1 min) and the microparticles were washed two times in tricine buffer, removing the buffer with centrifugation (3000 × g, 1 min). The samples were then mixed with 1 mL of 1 mg mL^–1^ PMA‐PDA in tricine buffer for 30 min and washed as with the PAH incubation. Next, 1 mL of liposomes with a concentration of 1 mg mL^–1^ total lipids was added to the microparticles, incubated for 30 min, and washed as with PMA‐PDA. This procedure was then repeated, alternating between PMA‐PDA and liposomes to deposit 3 bilayers of PMA‐PDA and liposomes onto the microparticles. Capsosomes were finally dispersed in tricine buffer (**Table** **1**).

**Table 1:** Capsosome configurations

Capsosome Name	Composition
SRB‐capsosomes	3× layers of SRB loaded liposomes (10 × 10^−3^ m srb)
SRB‐DiD‐capsosomes	Layer 1 and 2: SRB loaded liposomes (0.5 × 10^−3^ m srb), layer 3: DiD loaded liposomes (0.2 wt%)
DiD‐capsosomes	3× layers DiD loaded liposomes (1 wt%)
DiL‐capsosomes	3× layers DiL loaded liposomes (1 wt%)
Cap[VVV]	3× layers of vancomycin loaded liposomes
Cap[EEE]	3× layers of empty liposomes
Cap[VVE]	Layer 1 and 2: vancomycin loaded liposomes, layer 3: empty liposomes
Cap[EEP]	Layer 1 and 2: empty liposomes, layer 3: Palm‐Arg‐Arg‐NH_2_ loaded liposomes

For samples exposed to *S. aureus*, the capsosomes were prepared aseptically. The buffer and solutions of PAH, PMA‐PDA and liposomes were sterilized using a 0.45 µm filter (Corning, USA). The mesoporous silica microparticles were sonicated in 70 vol% ethanol for 10 min, and then washed 5× in 1 mL sterile tricine buffer using centrifugation (3000 × *g*, 1 min) to remove the supernatant.

### Bacterial Strains and Culture

The bacterial strains used are detailed in **Table** **2**.

**Table 2:** List of bacterial strains used

Strain	Description	Source
*Staphylococcus aureus* USA300 LAC JE2	A derivative of CA‐MRSA USA300 LAC, cured of plasmids	[63]
*Staphylococcus aureus* USA300 LAC	LAC strain of the USA CA‐MRSA lineage	[37]
*Staphylococcus aureus* USA300 LAC Δ*agrA*	USA300 with the *agrA* gene deleted	[40]
*Lactococcus lactis* NZ9800	*nisA* defective isogenic mutant of NZ9700 (*fnb‐*)	[64]

*S. aureus* JE2, *S. aureus* USA300, *S. aureus* USA300 *ΔagrA*, were plated on tryptic soy agar (TSA) from glycerol stocks. *L. lactis* was plated on M17 agar supplemented with 0.5 wt% glucose. A single colony from the plate was added to 5 mL of the appropriate media using an inoculation loop and incubated for 17 h at 37 °C in a shaking incubator (180 rpm).

For preparation of bacterial supernatants containing toxins, a single colony of *S. aureus* JE2, *S. aureus* USA300, *S. aureus* USA300 *ΔagrA*, or *L. lactis* was added to 10 mL of TSB using an inoculation loop and incubated for 16 h at 37 °C in a shaking incubator (180 rpm). Cultures were centrifuged at 6500 × g for 10 min at 4 °C. To separate the bacteria from the secreted toxins in the media, the cultures were filtered using a 0.45 µm filter.

### Toxin‐Triggered Release from SRB‐Capsosomes

For incubation of capsosomes with bacterial cultures, a 96‐well plate was prepared with column A containing 95 µL of 5 mg mL^–1^ SRB‐capsosomes and 95 µL TSB, and all other wells containing 95 µL TSB. A serial dilution was performed across the columns with the final column containing no capsosomes. Stationary‐phase cultures of *S. aureus* JE2, *S. aureus* USA300, and *S. aureus* USA300 *ΔagrA* were diluted and inoculated into the 96‐well plate to reach a final cell density of ≈1 × 10^5 ^CFU mL^–1^. For incubation of capsosomes with bacterial supernatants, a 96‐well plate was prepared containing 50 µL of 5 mg mL^–1^ SRB‐capsosomes and 50 µL buffer in column A, which was serially diluted into 50 µL buffer in all other wells as with the bacterial culture samples. A 50 µL aliquot of bacterial supernatant was then added to each well. For incubation of capsosomes with synthetic PSMs, 200 × 10^−6^ m synthetic PSMs were mixed with 0.05 mg SRB‐capsosomes in buffer to a final volume of 100 µL. Samples were measured every 30 min for 17 h using a Tecan Infinity 200 Pro plate reader with an excitation wavelength of 553 nm and an emission wavelength of 585 nm. Triton was then added to column A to form a final concentration of 1 vol%. The samples were incubated for 1 h shaking and then measured again using a plate reader.

### Bacterial Killing Assays to Test In Vitro Activity of Capsosomes

Capsosome samples (5 mg Cap[VVV] and Cap[EEE]; or 4 µg mL^–1^ free vancomycin) were resuspended in 95 µL tricine buffer. The 95 µL samples were mixed with 95 µL TSB and then 10 µL of diluted *S. aureus* JE2 culture was added to each sample to reach a cell density of ≈2 × 10^4^ CFU mL^‐1^. The samples were then incubated at 37 °C. For each timepoint (0, 2, 4, 6, 8, and 24 h) aliquots were taken and 10‐fold serial dilutions in PBS were prepared in a 96‐well plate from the neat sample down to 10^–7^. TSA plates were then split into eight sections and 10 µL of neat sample and each serial dilution was spread evenly onto the different sections. The plates were incubated at 37 °C for 17 h, after which the colonies were counted. For spot on plates 5 µL of the same dilutions were pipetted onto TSA plates. For Cap[VVP], Cap[EEP], and Cap[VVE] serial dilutions were prepared starting with 2.5 mg down to 0.3125 mg of capsosomes and from then samples were treated as described above and data of 1.25 mg is shown in Figure 4. The bacterial survival was calculated as a percentage of the number of bacteria in the starting inoculum: Survival / % = CFU_timepoint_/CFU_t0_ × 100.

### Bacterial Killing Assays to Test Synergy of Antibiotics

A *S. aureus* JE2 culture was adjusted to ≈2 × 10^6 ^CFU mL^–1^ and mixed 1:1 with antibiotics solution to reach a final concentration of 4 *μ*g mL^−1^. Samples were incubated at 37 °C and aliquots were taken after 0 and 8 h, serially diluted and plated on TSA plates as described above.

### Nanoparticle Tracking Analysis (NTA) to Measure Binding Capacity of Liposomes on Capsosomes

Liposome concentration was measured using a NanoSight NS300 (Malvern, UK). Samples were diluted (5000× dilution for 1 mg mL^–1^ lipid content, or equivalent) and applied to a continuous flow chamber at 50 µL min^–1^. A screen gain of 1 and camera level of 14 were used for measurements, which comprised 3–5 captures, each of which was 30–60 s. For analysis, a screen gain of 5.1 and a detection level of 10 were used.

### Analytical High Performance Liquid Chromatography (HPLC) to Analyze Vancomycin Loading

Samples containing vancomycin were analyzed with a 1260 Infinity LCMS (Agilent Technologies, USA) using an 8 min isocratic method in 20 × 10^−3^ m ammonium formate, 12% methanol pH 4, with an injection volume of 20 µL, a column temperature of 50 °C, and a flow rate of 1.5 mL min^–1^. Absorbance was measured at 240 nm. Peak area corresponding to vancomycin was measured using the integrate gadget in Origin (OriginLab, USA). A standard curve from 0 to 10 µg mL^–1^ vancomycin was constructed to determine vancomycin loading into liposomes.

### Liquid Chromatography Mass Spectrometry (LCMS) to Analyze Palm‐Arg‐Arg‐NH_2_ Loading

Samples containing Palm‐Arg‐Arg‐NH_2_ or PDA were analyzed with a 1260 Infinity LCMS (Agilent Technologies, USA) using an 10 min method with a 5 µL injection volume, consisting of 95% A (99.9 vol% dH_2_O, 0.1 vol% formic acid) 5% B (99.9 vol% acetonitrile, 0.1 vol% formic acid) from 0 to 0.5 min, a gradient to 5% A 95% B from 0.5 to 4.5 min, maintenance at 5% A 95% B from 4.5 to 6 min, a gradient to 95% A 5% B from 6 to 9 min, and maintenance at 95% A 5% B from 9 to 10 min. The flow rate was 0.5 mL min^–1^ and the column temperature was 40 °C. Absorbance was measured at 220 nm and species were detected using a 100–1000 Da method. For quantification of Palm‐Arg‐Arg‐NH_2_, ions with a m/z ratio corresponding to [M+H], [M+Na] or [M+2H], located between *x* = 4 min to *x* = 7 min, were extracted from the positive ion total ion current chromatogram to form an extracted ion chromatogram (EIC). Peak area of the EIC was calculated using the integrate gadget in Origin (OriginLab, USA). A standard curve from 0 to 15 µg mL^–1^ Palm‐Arg‐Arg‐NH_2_ was constructed to determine loading of Palm‐Arg‐Arg‐NH_2_ into liposomes.

### Flow Cytometry to Characterize Layer Deposition onto Capsosomes

Samples were characterized using a BD LSRFortessa flow cytometer with FACS DIVA software (BD Biosciences, USA). The samples were analyzed by measuring the FSC‐A, SSCA, DAPI fluorescence intensity (405 nm laser, 450/50 nm laser filter), and DiD fluorescence intensity (640 nm laser, 670/15 nm laser filter). For analyzing layer deposition onto capsosomes or, single capsosomes were defined using an FSC‐A versus SSC‐A gate drawn around the major population observed in each sample. Median fluorescence intensities were taken from a total of 20 000 single particle events. Data analysis was performed using FlowJo software (Tree Star, USA).

### Fluorescence Microscopy of Capsosomes and Bacteria

A 1 mL sample of stationary phase culture of *S. aureus* JE2 was harvested by centrifugation (13 500 rpm, 3 min) and washed in 1 mL PBS. The OD_600_ was adjusted to 1 in labelling buffer (50 × 10^−3^ m Na_2_CO_3_, 100 × 10^−3^ m NaCl, pH 8.0) with 0.5 mg mL^–1^ FITC and incubated for 30 min at room temperature. Labelled cells were washed 5× in 1 mL PBS, mixed 1:1 with 10 µL of 5 mg mL^–1^ SRB‐DiD‐capsosomes and incubated for 2 h. For imaging these samples, an 8 well µ‐Slide (ibidi, Germany) was covered with 10 mg mL^–1^ protamine solution and incubated for 30 min to coat the wells with protamine. For removal of excess protamine, the wells were washed 5× with PBS. The coating was performed to immobilize the bacteria to increase the quality of the microscopy. A 20 µL aliquot of the sample (or of just capsosomes) was then added to the 8 well µ‐Slide and imaged using a Nikon Ti Microscope with a 100× oil immersion objective. Z‐stacks were deconvolved using the EpiDEMIC plugin in Icy (50 iterations)^[^ ^65^ ^]^ and images were then processed (brightness/contrast adjustment) in Fiji.^[^ ^62^ ^]^

### Hemolysis Assay

Sheep blood was diluted to 8 vol% in PBS. Samples were prepared containing a 5 mg sample of Cap[VVV] or Cap[VVP], or 0–512 µg mL^–1^ free Palm‐Arg‐Arg‐NH_2_ and free vancomycin in 100 µL PBS and mixed with 100 µL 8 vol% sheep blood. Samples were incubated at 37 °C for 2 h and then centrifuged at 1577 g for 8 min. The supernatant (100 µL) was then transferred to a 96‐well plate and the absorbance measured at 570 nm in a plate reader. Hemolysis was calculated as follows

(1) $$\text{Haemolysis}\left(\%\right)=\frac{\mathrm{Ab}s_{x}-\mathrm{Ab}s_{-\mathrm{ve}}}{\mathrm{Ab}s_{+\mathrm{ve}}-\mathrm{Ab}s_{-\mathrm{ve}}}$$

where *Abs_x_* is the absorbance of the sample, *Abs*_−*ve*_ is the absorbance of the negative control (red blood cells with no treatment), and *Abs*_+*ve*_ is the absorbance of RBC lysed with 2 vol% Triton.

### In Vivo Activity of Capsosomes Tested in a *Drosophila melanogaster* Infection Model

Males of *w* ^1118^ *D. melanogaster* (fruit fly) were collected within 24–48 h following eclosion and kept in vials containing fresh fly food (tap water, 10 wt% Brewer's yeast, 2 wt% polenta, 8 wt% fructose, 0.8 wt% agar, 0.5 vol% nipagin, and 0.75 vol% propionic acid). Experiments were carried out using flies between 5 and 10 d old. The flies were injected intra‐abdominally with 50 nL fluorescently labelled capsosomes (DiL); *S. aureus* JE2 only (≈4 × 10^5^ CFU mL^–1^); buffer control, Cap[VVV] or [VVP] and *S. aureus* JE2 using a ratio of 5 mg capsosomes: ≈2 × 10^4^ CFU mL^–1^ bacteria in a total volume of 200 µL (set up in the same way as the in vitro study). Injections were completed using a pulled‐glass capillary needle and a Picospritzer injector system (Parker, USA). All the injections were performed in the same area between the thorax and abdomen of the flies (please see Movie 1, Supporting Information). The amount of injected liquid was adjusted using a graticule in the microscope to ensure injection of equal volumes of 50 nL per fly. In order to achieve this, two parameters were utilized: pressure and time of injection. Pressure was kept constant, but the time of injection was modulated for each needle specifically: the narrower it was, the longer the injection time and vice versa. Typical settings used were 30 psi and injection times between 16 and 25 ms, depending on the needle. Every sample requires a different needle, hence, the time parameter had to be adjusted for each needle separately. Control samples were also prepared including uninjected flies (untreated), flies injected only with TSB media, flies injected with *S. aureus* JE2 and Cap[EEE], and flies injected with *S. aureus* JE2 and buffer. Following injection, the flies were kept at 29 °C. Survival of *D. melanogaster* was monitored daily over 4–5 d, using *n* = 20 for each sample condition, and the flies were tipped into fresh food every 2–3 d. Toxicity of the capsosomes was also assessed by injecting 50 nL of 5 mg mL^1^ Cap[EEE] and Cap[VVP] and monitoring fly survival over 5 d. Calculation of statistical significance was carried out using the surv_diff and the pairwise_survdiff function (with a Bonferroni *p* value adjustment method) in R.

### Statistical Analysis

The specific sample sizes and statistical tests and post hoc analysis are specified in the figure captions. Data normalization and representation are also explained in each caption. All the data was plotted using Origin (OriginLab, USA). Statistical tests were performed in GraphPad Prism 9.0.0., except the fly survival curves were analyzed in R.

## Conflict of Interest

The authors declare no conflict of interest.

## Supporting information

Supporting Information

Supplemental Movie 1

## Data Availability

The data that support the findings of this study are available from the corresponding author upon reasonable request.

## References

[1] N. D. Friedman , E. Temkin , Y. Carmeli , Clin. Microbiol. Infect. 2016, 22, 416. 26706614 10.1016/j.cmi.2015.12.002

[2] S. S. Magill , J. R. Edwards , W. Bamberg , Z. G. Beldavs , G. Dumyati , M. A. Kainer , R. Lynfield , M. Maloney , L. McAllister‐Hollod , J. Nadle , S. M. Ray , D. L. Thompson , L. E. Wilson , S. K. Fridkin , N. Engl. J. Med. 2014, 370, 1198. 24670166 10.1056/NEJMoa1306801 PMC4648343

[3] A. Henderson , G. R. Nimmo , Br. Med. Bull. 2018, 125, 25. 29190327 10.1093/bmb/ldx046

[4] W. Zamoner , I. R. S. Prado , A. L. Balbi , D. Ponce , Clin. Exp. Pharmacol. Physiol. 2019, 46, 292. 30623980 10.1111/1440-1681.13066

[5] B. Sayin , S. Çaliş , B. Atilla , S. Marangoz , A. A. Hincal , J. Microencapsulation 2006, 23, 553. 16980276 10.1080/02652040600775632

[6] E. Cevher , Z. Orhan , L. Mülazimoǧlu , D. Şensoy , M. Alper , A. Yildiz , Y. Özsoy , Int. J. Pharm. 2006, 317, 127. 16624509 10.1016/j.ijpharm.2006.03.014

[7] F. Davani , M. Alishahi , M. Sabzi , M. Khorram , A. Arastehfar , K. Zomorodian , Mater. Sci. Eng., C 2021, 123, 111975. 10.1016/j.msec.2021.111975 33812603

[8] X. Yu , Q. Pan , Z. Zheng , Y. Chen , Y. Chen , S. Weng , L. Huang , RSC Adv. 2018, 8, 37424. 35557787 10.1039/c8ra06659k PMC9089331

[9] C. T. Johnson , M. C. P. Sok , K. E. Martin , P. P. Kalelkar , J. D. Caplin , E. A. Botchwey , A. J. García , Sci. Adv. 2019, 5, eaaw1228. 31114804 10.1126/sciadv.aaw1228 PMC6524983

[10] R. S. Kalhapure , N. Suleman , C. Mocktar , N. Seedat , T. Govender , J. Pharm. Sci. 2015, 104, 872. 25546108 10.1002/jps.24298

[11] S. Yang , X. Han , Y. Yang , H. Qiao , Z. Yu , Y. Liu , J. Wang , T. Tang , ACS Appl. Mater. Interfaces 2018, 10, 14299. 29633833 10.1021/acsami.7b15678

[12] D. Pornpattananangkul , L. Zhang , S. Olson , S. Aryal , M. Obonyo , K. Vecchio , C. M. Huang , L. Zhang , J. Am. Chem. Soc. 2011, 133, 4132. 21344925 10.1021/ja111110e PMC3062754

[13] Y. Wu , Z. Song , H. Wang , H. Han , Nat. Commun. 2019, 10, 4464. 31578336 10.1038/s41467-019-12233-2 PMC6775118

[14] M. Potter , A. Najer , A. Klöckner , S. Zhang , M. N. Holme , V. Nele , J. Che , L. Massi , J. Penders , C. Saunders , J. J. Doutch , A. M. Edwards , O. Ces , M. M. Stevens , ACS Nano 2020, 14, 17333. 33290039 10.1021/acsnano.0c07459 PMC7760217

[15] A. Lin , Y. Liu , X. Zhu , X. Chen , J. Liu , Y. Zhou , X. Qin , J. Liu , ACS Nano 2019, 13, 13965. 31730327 10.1021/acsnano.9b05766

[16] J. S. Ribeiro , E. A. F. Bordini , J. A. Ferreira , L. Mei , N. Dubey , J. C. Fenno , E. Piva , R. G. Lund , A. Schwendeman , M. C. Bottino , ACS Appl. Mater. Interfaces 2020, 12, 16006. 32180395 10.1021/acsami.9b22964 PMC7370252

[17] M. Otto , Curr. Opin. Microbiol. 2014, 17, 32. 24581690 10.1016/j.mib.2013.11.004 PMC3942668

[18] U. Nayanathara , S. S. Kermaniyan , G. K. Such , Macromol. Rapid Commun. 2020, 41, 2000298. 10.1002/marc.202000298 32686228

[19] L. Hosta‐Rigau , B. Stadler , Yan, E. C. N. , J. K. Heath , F. Aibericio , F. Caruso , Adv. Funct. Mater. 2010, 20, 59.

[20] L. Hosta‐Rigau , R. Chandrawati , E. Saveriades , P. D. Odermatt , A. Postma , F. Ercole , K. Breheney , K. L. Wark , B. Städler , F. Caruso , Biomacromolecules 2010, 11, 3548. 21090570 10.1021/bm101020e

[21] B. Städler , R. Chandrawati , K. Goldie , F. Caruso , Langmuir 2009, 25, 6725. 19505154 10.1021/la900213a

[22] C. Y. Yoo , J. S. Seong , S. N. Park , Colloids Surf., B 2016, 144, 99. 10.1016/j.colsurfb.2016.04.010 27085041

[23] Z. Drulis‐Kawa , A. Dorotkiewicz‐Jach , Int. J. Pharm. 2010, 387, 187. 19969054 10.1016/j.ijpharm.2009.11.033

[24] R. Chandrawati , L. Hosta‐Rigau , D. Vanderstraaten , S. A. Lokuliyana , B. Städler , F. Albericio , F. Caruso , ACS Nano 2010, 4, 1351. 20192233 10.1021/nn901843j

[25] J. W. Maina , J. J. Richardson , R. Chandrawati , K. Kempe , M. P. Van Koeverden , F. Caruso , Langmuir 2015, 31, 7776. 26155947 10.1021/acs.langmuir.5b01667

[26] M. Schwiering , A. Brack , R. Stork , N. Hellmann , Biochim. Biophys. Acta, Biomembr. 2013, 1828, 1962. 10.1016/j.bbamem.2013.04.005 23590994

[27] B. D. Henry , D. R. Neill , K. A. Becker , S. Gore , L. Bricio‐Moreno , R. Ziobro , M. J. Edwards , K. Mühlemann , J. Steinmann , B. Kleuser , L. Japtok , M. Luginbühl , H. Wolfmeier , A. Scherag , E. Gulbins , A. Kadioglu , A. Draeger , E. B. Babiychuk , Nat. Biotechnol. 2015, 33, 81. 25362245 10.1038/nbt.3037

[28] H. Wolfmeier , S. C. Mansour , L. T. Liu , D. Pletzer , A. Draeger , E. B. Babiychuk , R. E. W. Hancock , EBioMedicine 2018, 33, 211. 29936135 10.1016/j.ebiom.2018.06.016 PMC6085503

[29] M. Huseby , K. Shi , C. Kent Brown , J. Digre , F. Mengistu , S. S. Keun , G. A. Bohach , P. M. Schlievert , D. H. Ohlendorf , C. A. Earhart , J. Bacteriol. 2007, 189, 8719. 17873030 10.1128/JB.00741-07 PMC2168928

[30] D. S. Kohane , Biotechnol. Bioeng. 2007, 96, 203. 17191251 10.1002/bit.21301

[31] Y. Ma , C. Cortez‐Jugo , J. Li , Z. Lin , R. T. Richardson , Y. Han , J. Zhou , M. Björnmalm , O. M. Feeney , Q. Z. Zhong , C. J. H. Porter , A. K. Wise , F. Caruso , Biomacromolecules 2019, 20, 3425. 31411865 10.1021/acs.biomac.9b00710

[32] Y. Ma , M. Björnmalm , A. K. Wise , C. Cortez‐Jugo , E. Revalor , Y. Ju , O. M. Feeney , R. T. Richardson , E. Hanssen , R. K. Shepherd , C. J. H. Porter , F. Caruso , ACS Appl. Mater. Interfaces 2018, 10, 31019. 30192499 10.1021/acsami.8b10415

[33] B. Städler , R. Chandrawati , A. D. Price , S.‐F. Chong , K. Breheney , A. Postma , L. A. Connal , A. N. Zelikin , F. Caruso , Angew. Chem., Int. Ed. 2009, 48, 4359. 10.1002/anie.200900386 19418505

[34] S. Mornet , O. Lambert , E. Duguet , A. Brisson , Nano Lett. 2005, 5, 281. 15794611 10.1021/nl048153y

[35] O. Kulygin , A. D. Price , S. F. Chong , B. Städler , A. N. Zelikin , F. Caruso , Small 2010, 6, 1558. 20578114 10.1002/smll.201000453

[36] W. Bae , T. Y. Yoon , C. Jeong , PLoS One 2021, 16, 0247326. 10.1371/journal.pone.0247326 PMC7895399 33606817

[37] B. A. Diep , S. R. Gill , R. F. Chang , T. H. Van Phan , J. H. Chen , M. G. Davidson , F. Lin , J. Lin , H. A. Carleton , E. F. Mongodin , G. F. Sensabaugh , F. Perdreau‐Remington , Lancet 2006, 367, 731. 16517273 10.1016/S0140-6736(06)68231-7

[38] K. Ohlsen , K. P. Koller , J. Hacker , Infect. Immun. 1997, 65, 3606. 9284126 10.1128/iai.65.9.3606-3614.1997 PMC175513

[39] C. Jenul , A. R. Horswill , Microbiol. Spectr. 2019, 7, 2. 10.1128/microbiolspec.gpp3-0031-2018 PMC6452892 30953424

[40] K. L. Painter , PhD thesis, The Molecular Basis of Persistent Staphylococcal Bacteraemia , Imperial College London, London 2015.

[41] M. Laabei , W. D. Jamieson , R. C. Massey , A. T. A. Jenkins , PLoS One 2014, 9, e87270. 24498061 10.1371/journal.pone.0087270 PMC3907525

[42] C. Watanakunakorn , Mode of Action and In‐Vitro Activity of Vancomycin, Vol. 14, Oxford Academic, Oxford 1984, pp. 7–18. 10.1093/jac/14.suppl_d.7 6440886

[43] N. Alshehri , A. E. Ahmed , N. Yenugadhati , S. Javad , K. Al Sulaiman , H. M. Al‐Dorzi , M. Aljerasiy , M. Badri , Ther. Clin. Risk Manage. 2020, 16, 979. 10.2147/TCRM.S266295 PMC7569025 33116547

[44] A. Hassoun , P. K. Linden , B. Friedman , Crit. Care 2017, 21, 211. 28807042 10.1186/s13054-017-1801-3 PMC5557425

[45] K. Uda , J. Suwa , K. Ito , H. Hataya , Y. Horikoshi , J. Pediatr. Pharmacol. Ther. 2019, 24, 450. 31598110 10.5863/1551-6776-24.5.450 PMC6782114

[46] M. Vestergaard , B. Leng , J. Haaber , M. S. Bojer , C. S. Vegge , H. Ingmer , Front. Microbiol. 2016, 7, 2018. 28066345 10.3389/fmicb.2016.02018 PMC5165250

[47] P. D. Tamma , S. E. Cosgrove , L. L. Maragakis , Clin. Microbiol. Rev. 2012, 25, 450. 22763634 10.1128/CMR.05041-11 PMC3416487

[48] R. C. Moellering , Am. J. Med. 1983, 75, 4. 10.1016/0002-9343(83)90088-8 6351605

[49] L. K. Hidayat , D. I. Hsu , R. Quist , K. A. Shriner , A. Wong‐Beringer , Arch. Intern. Med. 2006, 166, 2138. 17060545 10.1001/archinte.166.19.2138

[50] P. A. Moise , J. J. Schentag , Int. J. Antimicrob. Agents 2000, 16, 31. 11137406 10.1016/s0924-8579(00)00303-4

[51] C. L. Moore , M. Lu , F. Cheema , P. Osaki‐Kiyan , M. B. Perri , S. Donabedian , N. Z. Haque , M. J. Zervos , Antimicrob. Agents Chemother. 2011, 55, 4581. 21825294 10.1128/AAC.00115-11 PMC3186992

[52] C. Pinese , S. Jebors , C. Echalier , P. Licznar‐Fajardo , X. Garric , V. Humblot , C. Calers , J. Martinez , A. Mehdi , G. Subra , Adv. Healthcare Mater. 2016, 5, 3067. 10.1002/adhm.201600757 27792296

[53] C. D. Doern , J. Clin. Microbiol. 2014, 52, 4124. 24920779 10.1128/JCM.01121-14 PMC4313275

[54] B. El Shazely , G. Yu , P. R. Johnston , J. Rolff , Front. Microbiol. 2020, 11, 103. 32117132 10.3389/fmicb.2020.00103 PMC7033599

[55] M. S. Zharkova , D. S. Orlov , O. Y. Golubeva , O. B. Chakchir , I. E. Eliseev , T. M. Grinchuk , O. V. Shamova , Front. Cell. Infect. Microbiol. 2019, 9, 128. 31114762 10.3389/fcimb.2019.00128 PMC6503114

[56] L. T. Reiter , L. Potocki , S. Chien , M. Gribskov , E. Bier , Genome Res. 2001, 11, 1114. 11381037 10.1101/gr.169101 PMC311089

[57] C. J. Evans , V. Hartenstein , U. Banerjee , Dev. Cell 2003, 5, 673. 14602069 10.1016/s1534-5807(03)00335-6

[58] A. J. Needham , M. Kibart , H. Crossley , P. W. Ingham , S. J. Foster , Microbiology 2004, 150, 2347. 15256576 10.1099/mic.0.27116-0

[59] T. R. Nimal , G. Baranwal , M. C. Bavya , R. Biswas , R. Jayakumar , ACS Appl. Mater. Interfaces 2016, 8, 22074. 27508491 10.1021/acsami.6b07463

[60] M. Lelle , K. Peneva , Amino Acids 2014, 46, 1243. 24504931 10.1007/s00726-014-1685-3

[61] P. A. Parmar , S. C. Skaalure , L. W. Chow , J. P. St‐Pierre , V. Stoichevska , Y. Y. Peng , J. A. Werkmeister , J. A. M. Ramshaw , M. M. Stevens , Biomaterials 2016, 99, 56. 27214650 10.1016/j.biomaterials.2016.05.011 PMC4910873

[62] J. Schindelin , I. Arganda‐Carreras , E. Frise , V. Kaynig , M. Longair , T. Pietzsch , S. Preibisch , C. Rueden , S. Saalfeld , B. Schmid , J. Y. Tinevez , D. J. White , V. Hartenstein , K. Eliceiri , P. Tomancak , A. Cardona , Nat. Methods 2012, 9, 676. 22743772 10.1038/nmeth.2019 PMC3855844

[63] P. D. Fey , J. L. Endres , V. K. Yajjala , T. J. Widhelm , R. J. Boissy , J. L. Bose , K. W. Bayles , mBio 2013, 4, 00537. 10.1128/mBio.00537-12 PMC3573662 23404398

[64] O. P. Kuipers , M. M. Beerthuyzen , R. J. Siezen , W. M. De Vos , Eur. J. Biochem. 1993, 216, 281. 7689965 10.1111/j.1432-1033.1993.tb18143.x

[65] F. De Chaumont , S. Dallongeville , N. Chenouard , N. Hervé , S. Pop , T. Provoost , V. Meas‐Yedid , P. Pankajakshan , T. Lecomte , Y. Le Montagner , T. Lagache , A. Dufour , J. C. Olivo‐Marin , Nat. Methods 2012, 9, 690. 22743774 10.1038/nmeth.2075

