# Supplementary material for: Bacterial Toxin-Triggered Release of Antibiotics from Capsosomes Protects a Fly Model from Lethal Methicillin-Resistant Staphylococcus aureus (MRSA) Infection
Source: Adv Healthc Mater. Author manuscript; Available in PMC 2024 Jan 6. (PMC7615487; doi:10.1002/adhm.202200036)
Supplement: Supplementary Materials [file EMS175895-supplement-Supplementary_Materials.pdf]

ADVANCED HEALTHCARE MATERIALS

Supporting Information

for *Adv. Healthcare Mater.*, DOI 10.1002/adhm.202200036

Bacterial Toxin-Triggered Release of Antibiotics from Capsosomes Protects a Fly Model from Lethal Methicillin-Resistant *Staphylococcus aureus* (MRSA) Infection

Renée L. Tonkin, Anna Klöckner, Adrian Najer, Carolina J. Simoes da Silva, Cécile Echalier, Marc S. Dionne, Andrew M. Edwards and Molly M. Stevens**

Supporting Information

Bacterial toxin-triggered release of antibiotics from capsosomes protects a fly model from lethal methicillin-resistant *Staphylococcus aureus* (MRSA) infection

Renée L. Tonkin,[†] *Anna Klöckner,*[†] *Adrian Najer,*[†] *Carolina J. Simoes Da Silva, Cécile Echalier, Marc S. Dionne, Andrew M. Edwards*[*] *and Molly M. Stevens*[*]

Supplementary Figures

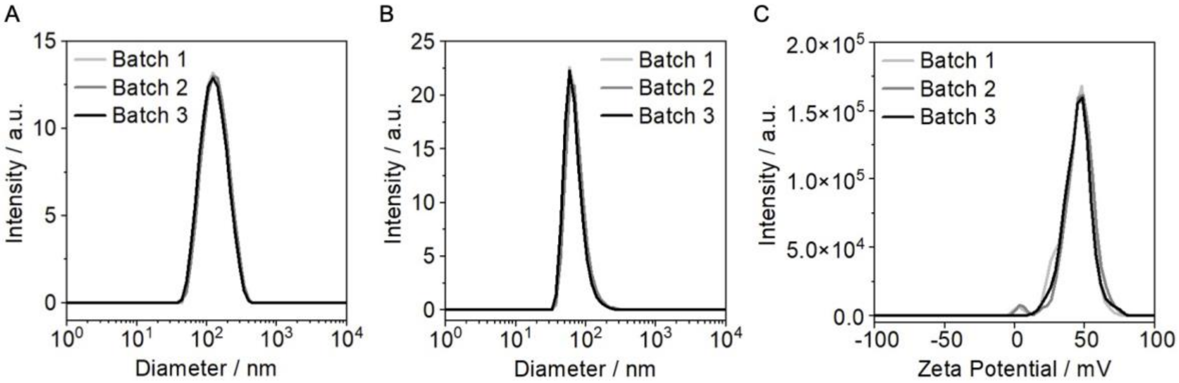

Supplementary Figure 1: Liposome Characterisation. (A) Intensity distribution of liposome diameter measured by DLS. Mean ± SD = 140 ± 59 nm (N = 3, n = 3). (B) Number distribution of liposome diameter measured by DLS. Mean ± SD = 72 ± 3 nm (N = 3, n = 3). (C) Zeta potential of liposomes. Mean ± SD = +45 ± 10 mV (N = 3, n = 3).

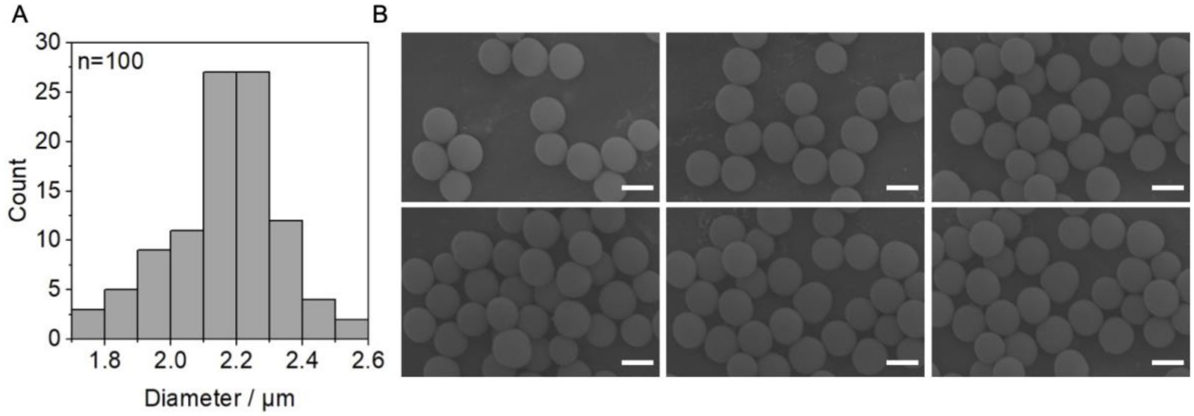

Supplementary Figure S2: Diameter of mesoporous silica microparticles. (A) Histogram of particle diameters measured from SEM images. Mean ± SD = 2.2 ± 0.2 µm (N = 100). (B) SEM images of mesoporous silica microparticles used for diameter analysis. Scale bars = 2 µm.

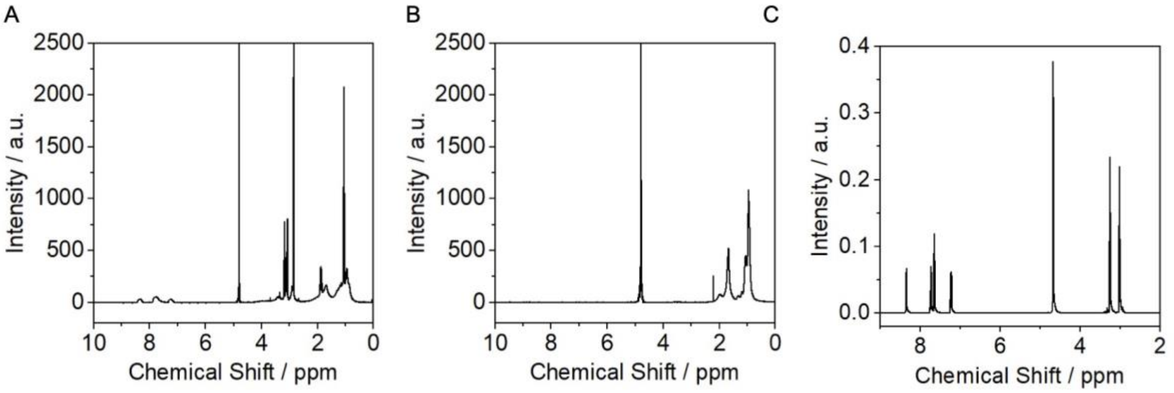

Supplementary Figure S3: ^{1}H NMR of (A) PMA-PDA, (B) PMA, and (C) PDA in D_2O. The additional chemical shifts (δ = 0.93-0.99 (t), 1.74-1.83 (p), 2.7-2.8 (s), 2.94-3.15 (m)) in (A) were attributed to 1-(3-dimethylaminopropyl)-3-ethylurea (EDU), a urea derivative of EDC.

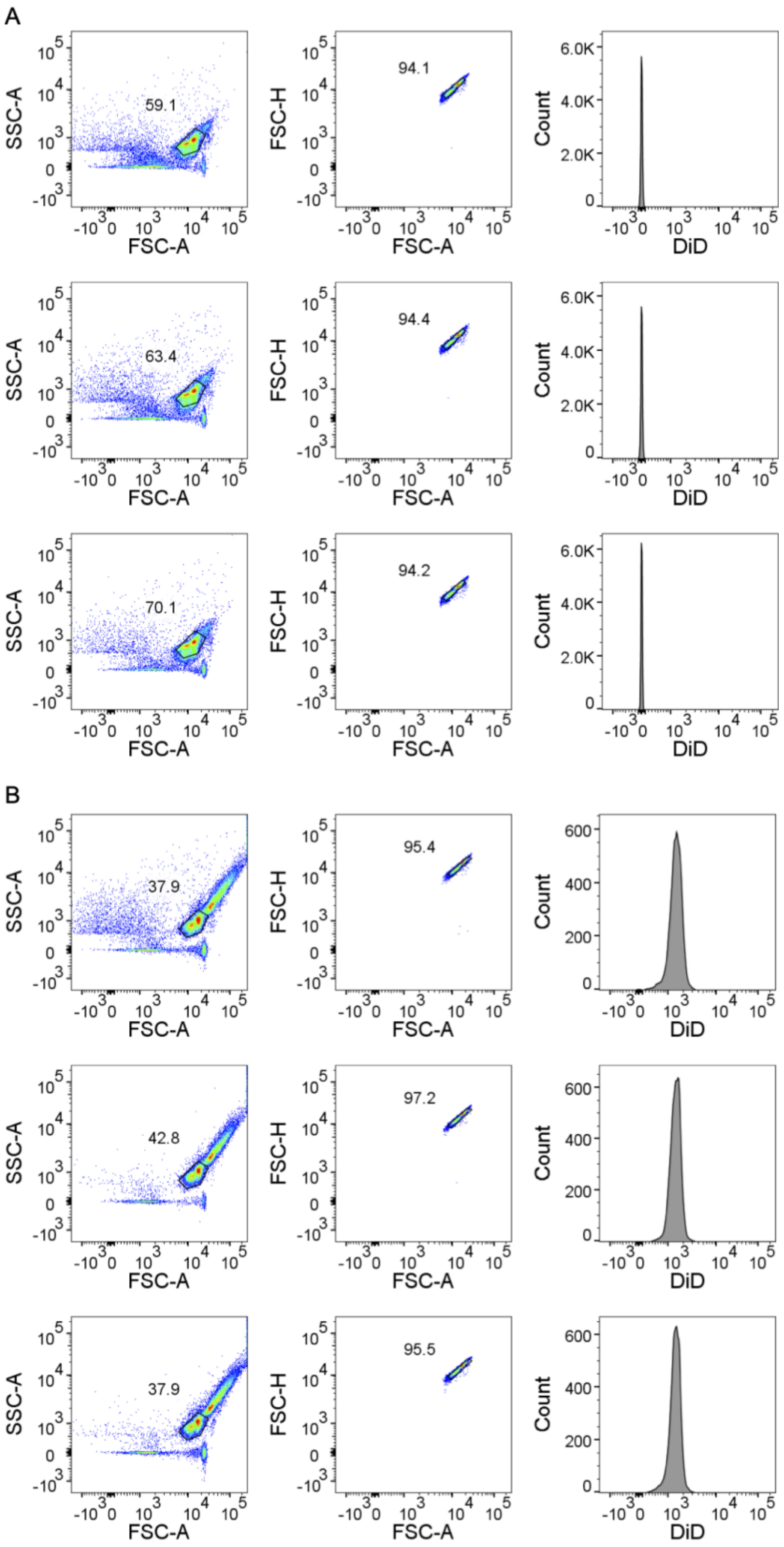
A
SSC-A
FSC-A
FSC-H
Count
DiD
59.1
94.1
63.4
94.4
70.1
94.2
6.0K
4.0K
2.0K
0
B
37.9
95.4
42.8
97.2
37.9
95.5
600
400
200
0

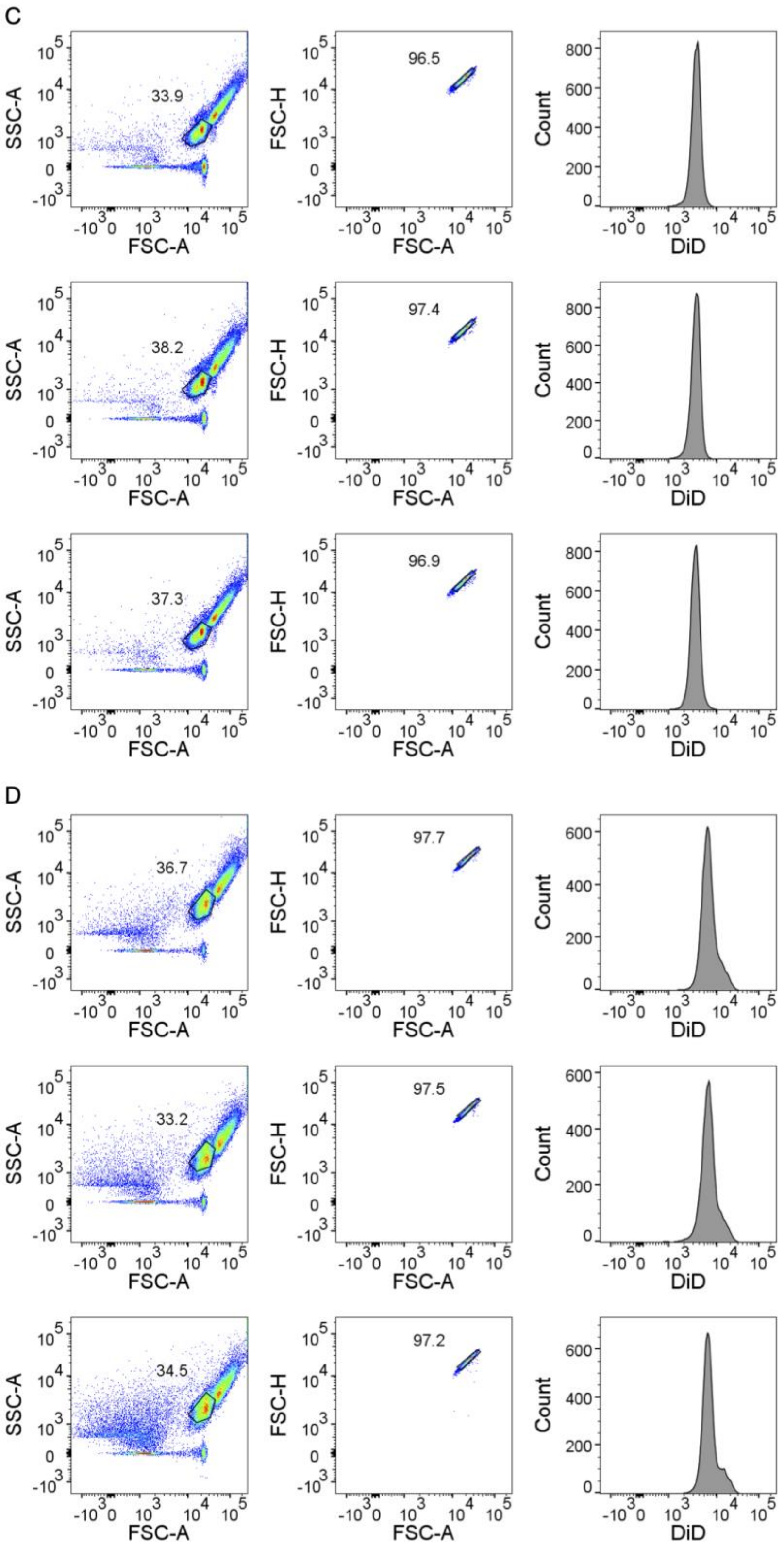

Supplementary Figure S4: Flow cytometry of layer-by-layer assembly of capsosomes. (A) No layers of liposomes, (B) one layer of DiD-loaded liposomes, (C) two layers of DiD-loaded liposomes, (D) three layers of DiD-loaded liposomes.

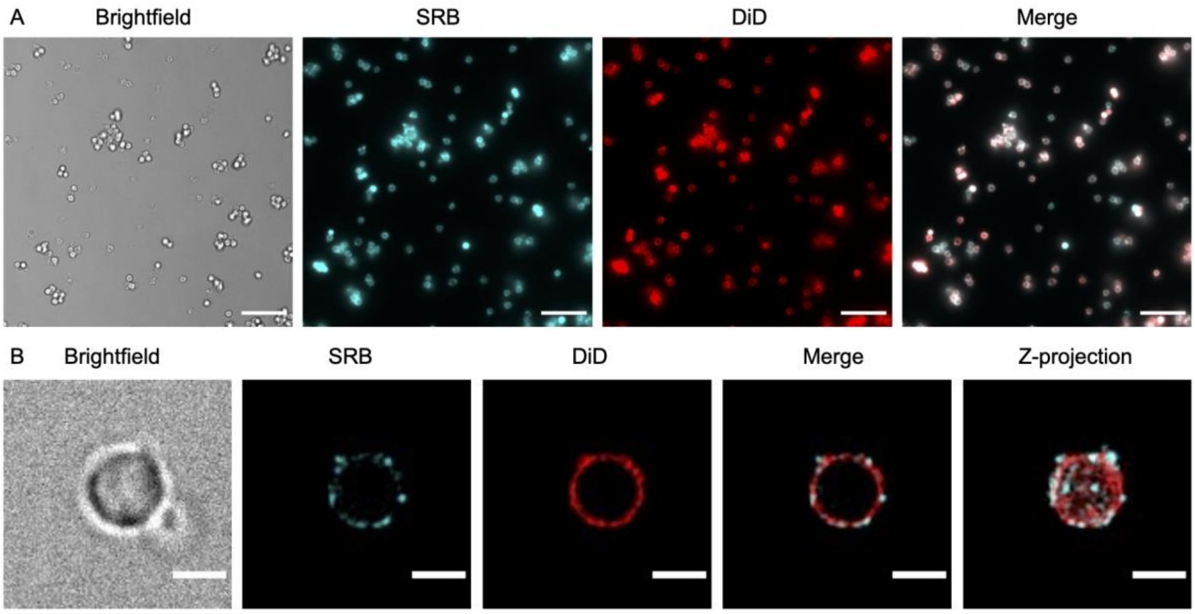

Supplementary Figure S5: Loading of hydrophilic and hydrophobic cargoes in capsosomes. (A) Widefield fluorescence micrographs showing an overview of capsosomes containing SRB-loaded liposomes in layers 1 and 2 and DiD-loaded liposomes in layer 3. Scale bars: 20 µm. (B) Deconvolved widefield fluorescence micrographs showing an individual capsosome containing SRB-loaded liposomes in layers 1 and 2 and DiD-loaded liposomes in layer 3. Scale bars: 2 µm.

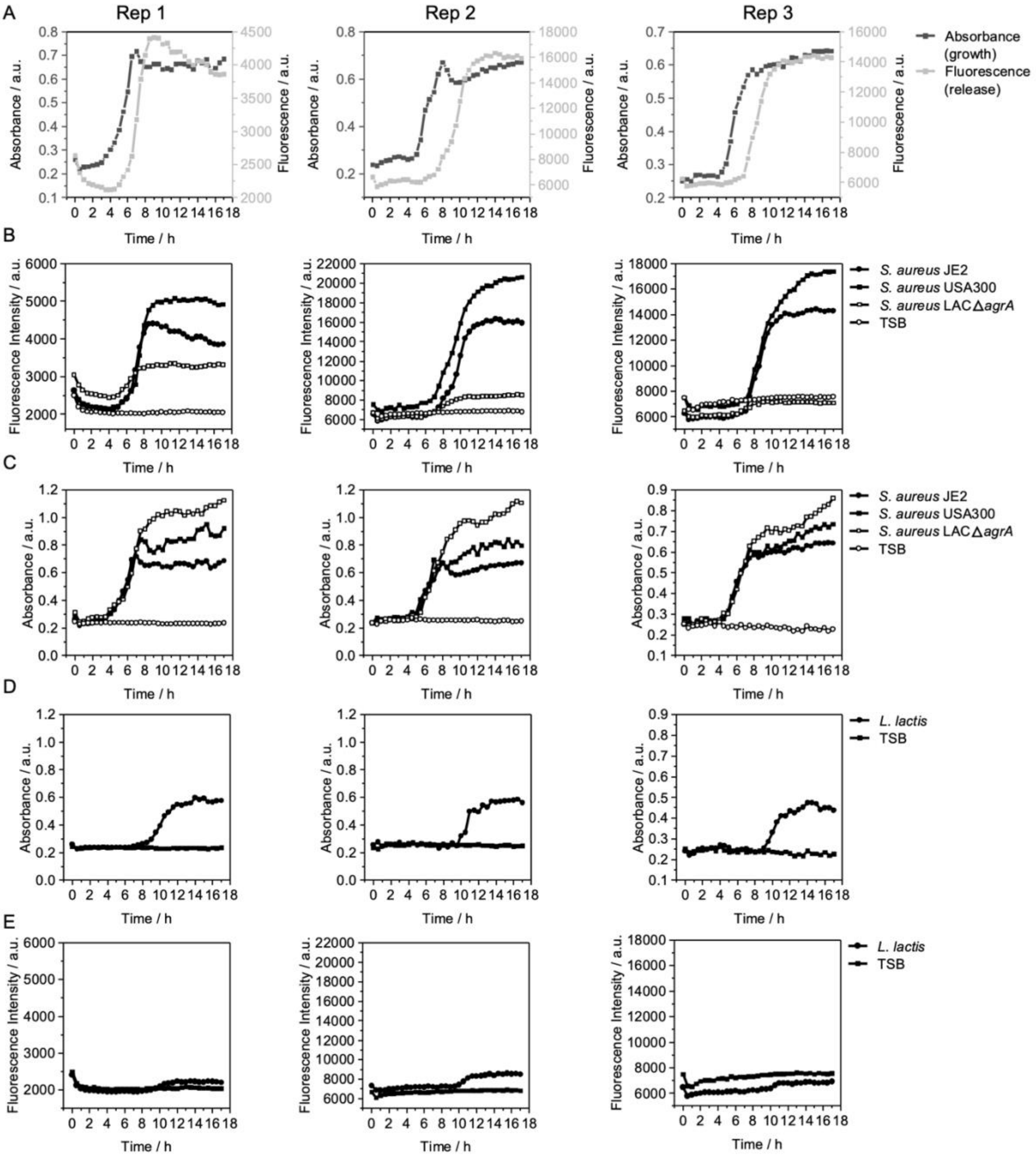

Supplementary Figure S6: Toxin-triggered release of sulforhodamine B (SRB) from capsosomes. (A) Absorbance (indicating bacterial growth) and fluorescence intensity (indicating SRB release) of SRB-loaded capsosomes and *S. aureus* JE2 over 17 h. (B) Fluorescence intensity of capsosomes with *S. aureus* JE2 (from A), *S. aureus* USA300, *S. aureus* Δ*agr* mutant (low toxin expression), and TSB over 17 h. (C) Absorbance of capsosomes with *S. aureus* JE2, *S. aureus* USA300, *S. aureus* Δ*agr* mutant (low toxin expression), and TSB over 17 h. (D) Absorbance of capsosomes with *L. lactis* and TSB (from C) over 17 h. (E) Fluorescence intensity of capsosomes with *L. lactis* and TSB (from B) over 17 h. All absorbance measurements were taken at 600 nm. All fluorescence measurements were taken at 585 nm. Max-min normalized versions of this data set are represented in the main Figure 2.

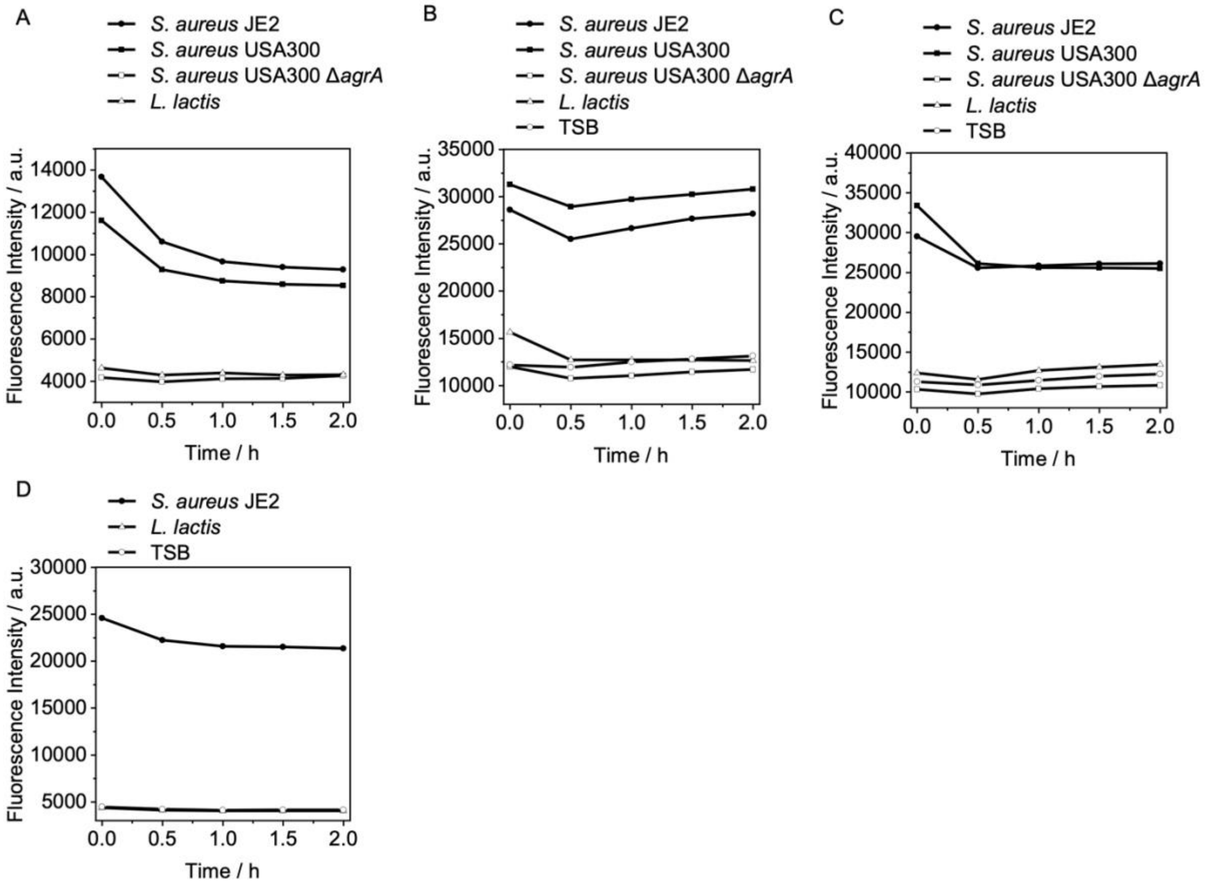

Supplementary Figure S7: Release by soluble secreted toxins. Fluorescence intensity of SRB-capsosomes with bacterial supernatants from *S. aureus* JE2, *S. aureus* USA300, *S. aureus* Δ*agr* mutant (low toxin expression), *L. lactis*, and TSB over 2 h after supernatant addition. A max-min normalized version of this data set is represented in the main Figure 2.

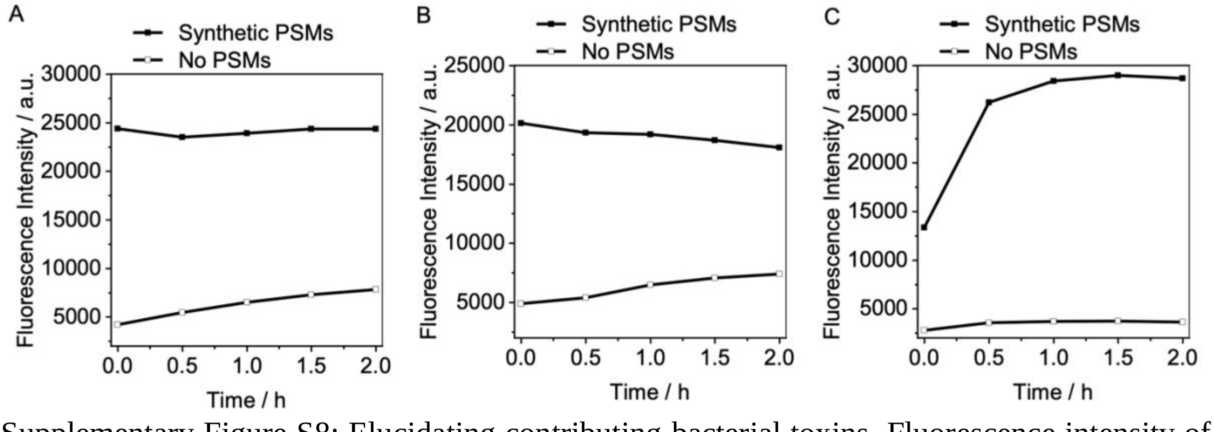

Supplementary Figure S8: Elucidating contributing bacterial toxins. Fluorescence intensity of SRB-capsosomes with and without synthetic PSMs over 2 h after PSM addition. A max-min normalized version of this data set is represented in the main Figure 2.

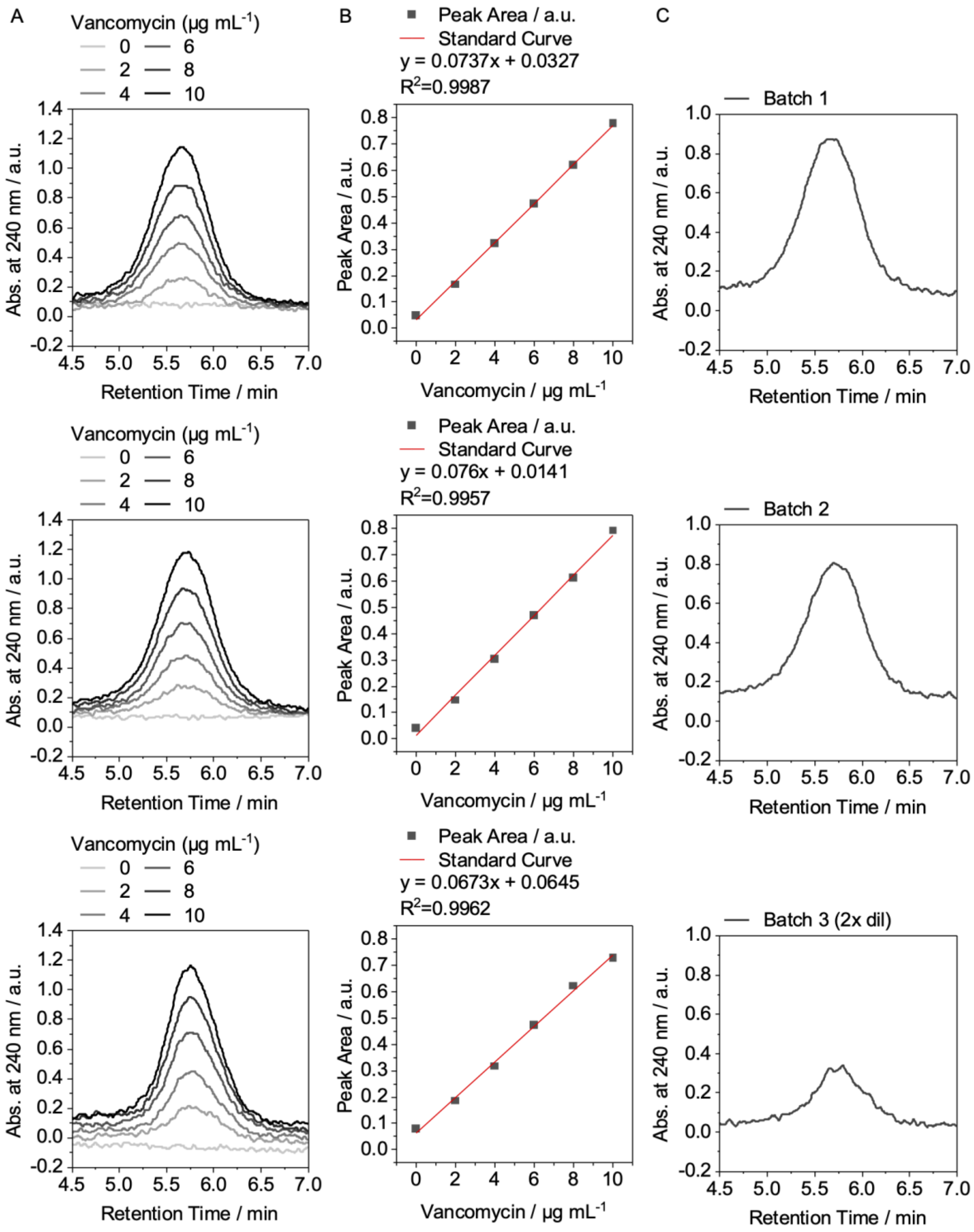

Supplementary Figure S9: Quantification of vancomycin loading. (A) Absorbance at 240 nm of standards containing 0-10 μg mL^{-1} vancomycin for batch repeats 1, 2 and 3. (B) Standard curves generated using the integrated peak areas for batch repeats 1, 2, and 3. (C) Absorbance at 240 nm of vancomycin loaded in liposomes for batch repeats 1, 2, and 3.

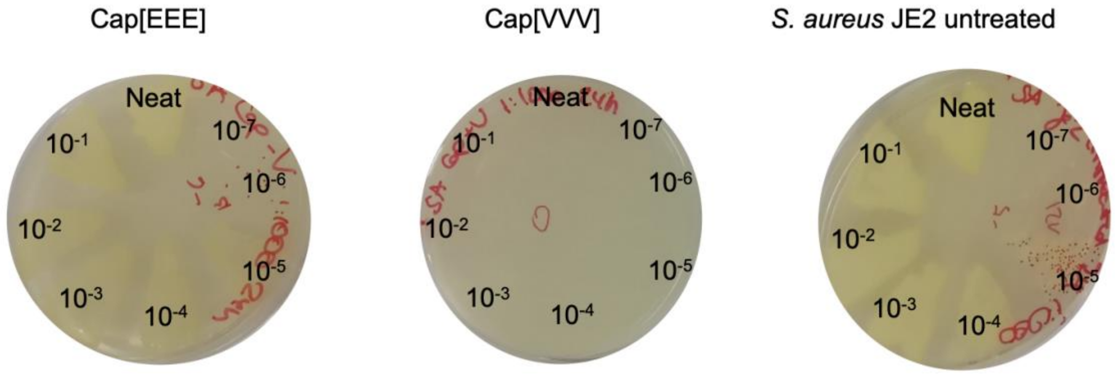

Supplementary Figure S10: Calculating killing efficacy of vancomycin-loaded capsosomes. Representative plated dilutions of *S. aureus* with empty capsosomes (Cap[EEE]), vancomycin-loaded capsosomes (Cap[VVV]), and untreated *S. aureus* JE2 at 24 h timepoint.

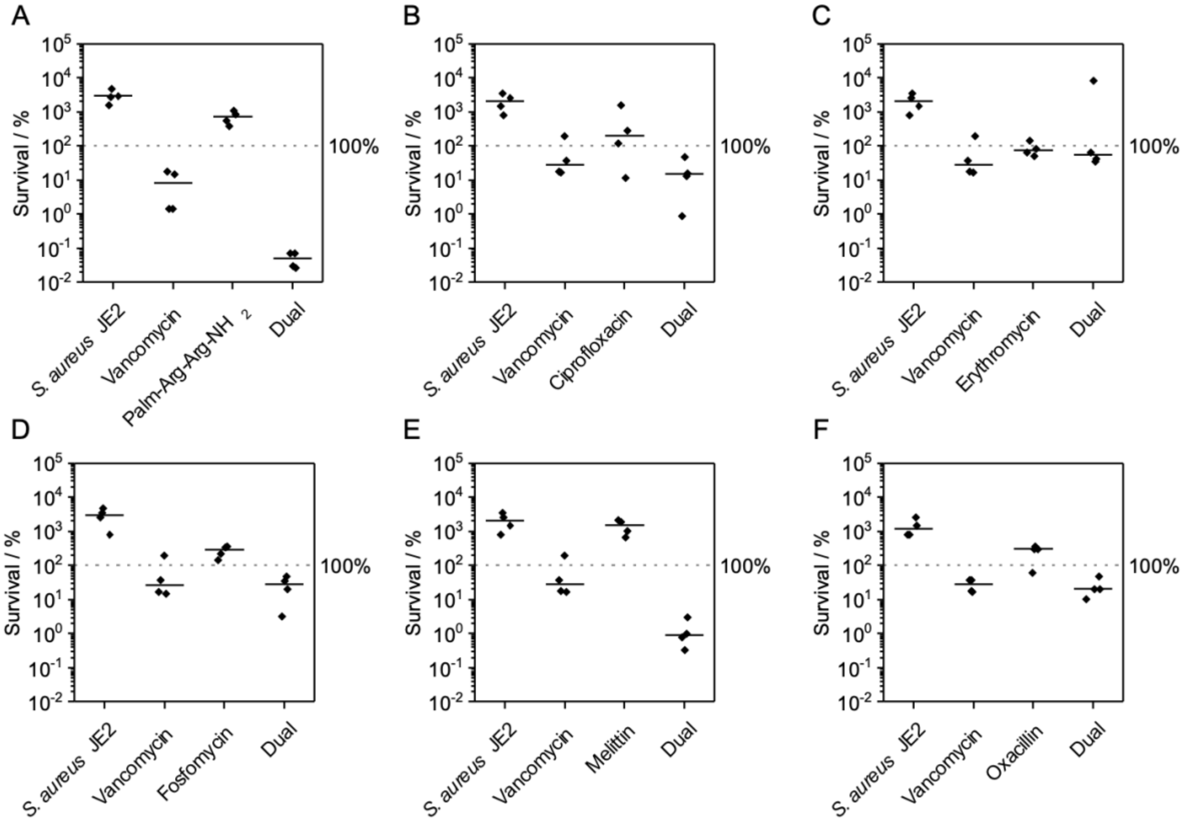

Supplementary Figure S11: Screening of antibacterial compounds with vancomycin to test for synergy. Vancomycin in combination with Palm-Arg-Arg-NH_2 (A), ciprofloxacin (B), erythromycin (C), fosfomycin (D), melittin (E), and oxacillin (F) against *S. aureus* JE2 (N = 4, n = 1, Oxacillin: N = 3, n = 1). Concentration of each antibiotic was 4 µg mL^{-1} and samples were incubated for 8 h with around 2 x 10^6 CFU mL^{-1} bacteria.

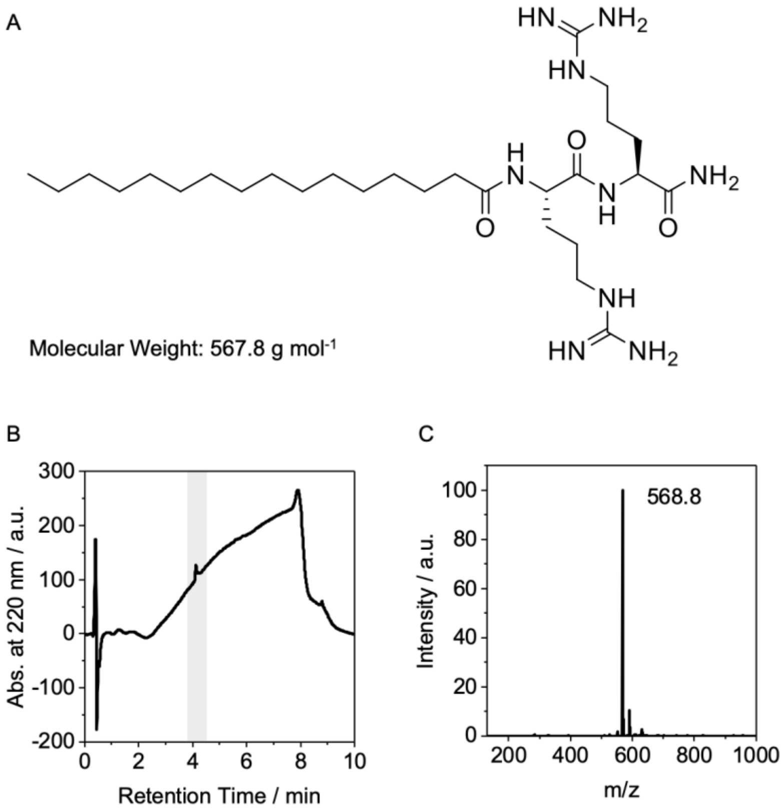

Supplementary Figure S12: Synthesis of Palm-Arg-Arg-NH_2. (A) Structure of Palm-Arg-Arg-NH_2. (B) HPLC of purified Palm-Arg-Arg-NH_2. (C) Mass spectrum corresponding to highlighted retention time in (B) showing [M+H] of 568.8, indicative of Palm-Arg-Arg-NH_2.

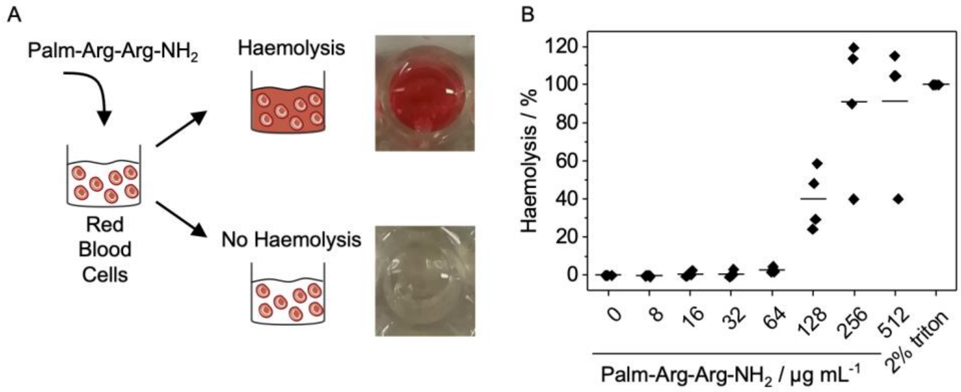

Supplementary Figure S13: Hemolysis assay of Palm-Arg-Arg-NH_2. (A) Schematic describing hemolysis assay. (B) Hemolytic effect of 0-512 µg mL^{-1} Palm-Arg-Arg-NH_2 on sheep RBCs (N = 4, n = 1). Percentage hemolysis was calculated by max-min normalization of data to the background untreated signal and complete cell lysis with 2% Triton. Lines indicate mean values.

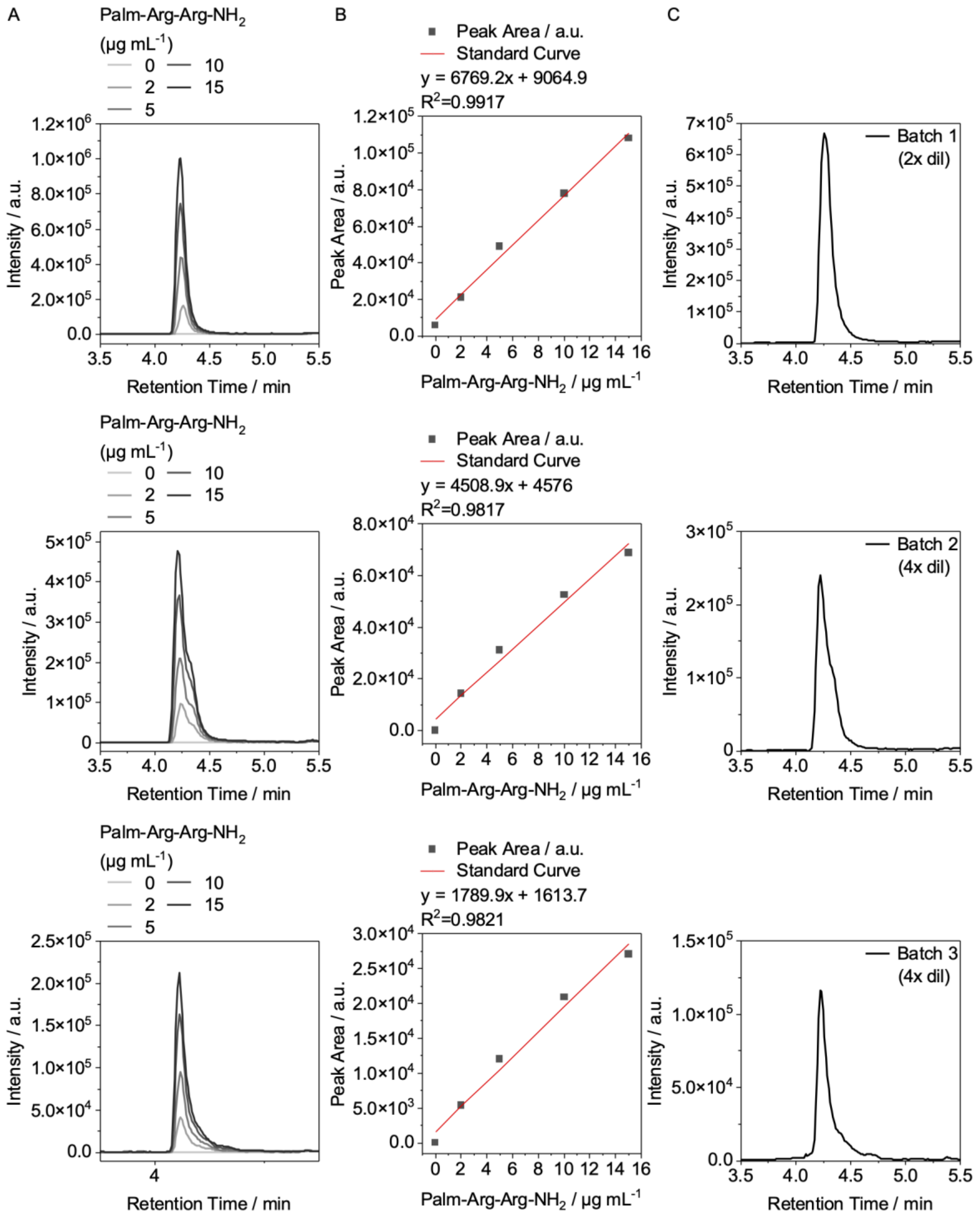

Supplementary Figure S14: Quantification of Palm-Arg-Arg-NH_2 loading. (A) Extracted ion chromatograms of standards containing 0-15 μg mL^{-1} Palm-Arg-Arg-NH_2 for batch repeats 1, 2 and 3. (B) Standard curves generated using the integrated peak areas for batch repeats 1, 2, and 3. (C) Extracted ion chromatograms of Palm-Arg-Arg-NH_2 loaded in liposomes for batch repeats 1, 2, and 3.

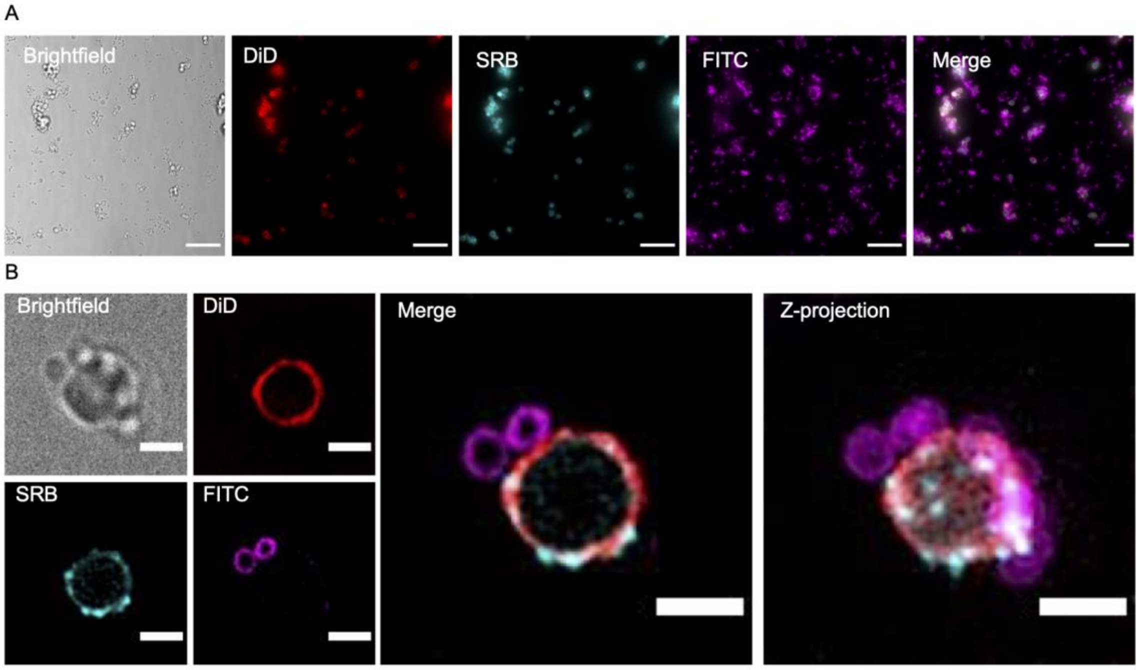

Supplementary Figure S15: Interaction between capsosomes and *S. aureus*. (A) Widefield fluorescence micrographs showing FITC-labelled *S. aureus* JE2 incubated with SRB-DiD-capsosomes (SRB in layers 1 and 2, DiD in layer 3). Scale bars: 20 µm. (B) Deconvolved widefield fluorescence micrographs showing individual SRB-DiD-capsosomes interacting with FITC-labelled *S. aureus* JE2. Scale bars: 2 µm.

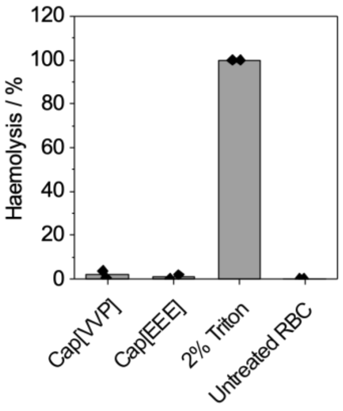

Supplementary Figure S16: Hemolytic toxicity of dual-loaded capsosomes. Hemolytic effect of Cap[VVP] and Cap[EEE] on sheep RBCs (N = 2, n = 1). Percentage hemolysis was calculated by max-min normalization of data to the background untreated signal and complete cell lysis with 2% Triton.

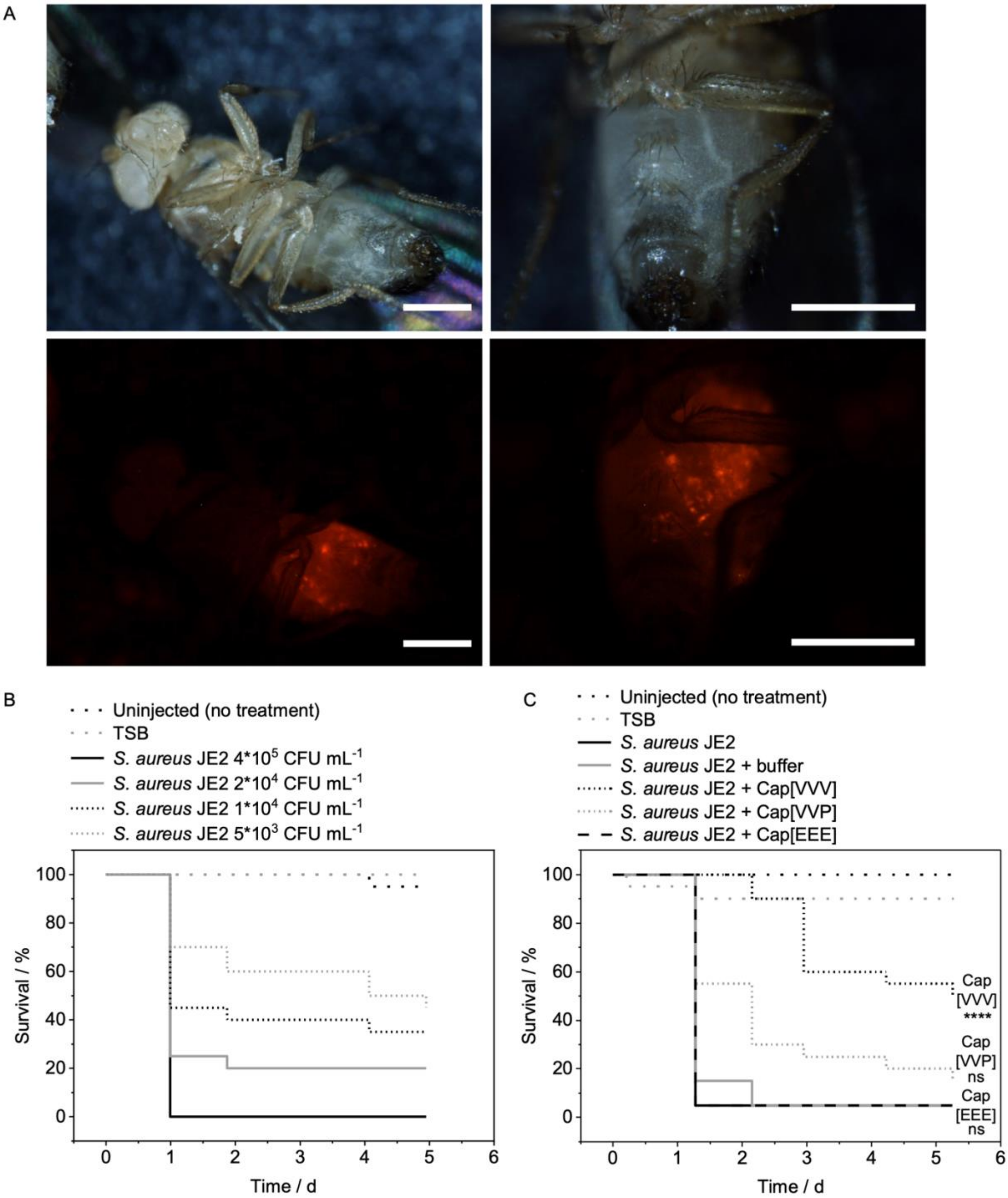

Supplementary Figure S17: (A) Zoomed images of DiL-capsosome distribution after injection in flies. Scale bars: 200 µm. (B) Correlation of fly survival with *S. aureus* JE2 inoculum concentration. (C) Survival of flies infected with *S. aureus* JE2 and treated with Cap[VVV], Cap[VVP] and Cap[EEE], or buffer control. Additionally, control groups of flies were left uninjected or uninfected (N = 1, n = 20).

Supplementary Movie 1: Example video showing the injection procedure in flies. A model blue dye was injected for visualization purposes.
